# Blocking Polyphosphate Mobilization Inhibits Pho4 Activation and Virulence in the Pathogen Candida albicans

**DOI:** 10.1128/mbio.00342-22

**Published:** 2022-05-16

**Authors:** Yasmin Ahmed, Mélanie A. C. Ikeh, Donna M. MacCallum, Alison M. Day, Kevin Waldron, Janet Quinn

**Affiliations:** a Biosciences Institute, Faculty of Medical Sciences, Newcastle University, Newcastle upon Tyne, United Kingdom; b Aberdeen Fungal Group, Institute of Medical Sciences, University of Aberdeen, Aberdeen, United Kingdom; University of British Columbia

**Keywords:** *Candida albicans*, morphogenesis, phosphate metabolism, stress response, virulence

## Abstract

The ability of pathogenic fungi to obtain essential nutrients from the host is vital for virulence. In Candida albicans, acquisition of the macronutrient phosphate is regulated by the Pho4 transcription factor and is important for both virulence and resistance to host-encountered stresses. All cells store phosphate in the form of polyphosphate (polyP), a ubiquitous polymer comprising tens to hundreds of phosphate residues. Release of phosphate from polyP is one of the first responses evoked in response to phosphate starvation, and here, we sought to explore the importance of polyP mobilization in the pathobiology of C. albicans. We found that two polyphosphatases, Ppn1 and Ppx1, function redundantly to release phosphate from polyP in C. albicans. Strikingly, we reveal that blocking polyP mobilization prevents the activation of the Pho4 transcription factor: following P_i_ starvation, Pho4 fails to accumulate in the nucleus and induce P_i_ acquisition genes in *ppn1*Δ *ppx1*Δ cells. Consequently, *ppn1*Δ *ppx1*Δ cells display impaired resistance to the same range of stresses that require Pho4 for survival. In addition, cells lacking both polyphosphatases are exquisitely sensitive to DNA replication stress, indicating that polyP mobilization is needed to support the phosphate-demanding process of DNA replication. Blocking polyP mobilization also results in significant morphological defects, as *ppn1*Δ *ppx1*Δ cells form large pseudohypha-like cells that are resistant to serum-induced hypha formation. Thus, polyP mobilization impacts key processes important for the pathobiology of C. albicans, and consistent with this, we found that blocking this process attenuates the virulence of this important human fungal pathogen.

## INTRODUCTION

Candida albicans is a gut commensal and opportunistic fungal pathogen of humans. In immunocompromised hosts, this fungus can bypass diminished mucosal immune defenses and enter the bloodstream, resulting in systemic infections which are associated with an alarming mortality rate of over 40% (1). One notable characteristic of C. albicans is its ability to colonize multiple anatomical niches within the host, both as a commensal organism and during systemic infections. A key driver in promoting C. albicans colonization of diverse host environments is its metabolic flexibility, which allows the acquisition of essential nutrients in resource-poor settings and in complex microenvironments where competition for nutrients is high (2). Sophisticated mechanisms are in place to allow the acquisition of essential micronutrients, exemplified by secretion of a scavenger protein, Pra1, which sequesters zinc from host cells before reassociating with the fungal cell via the zinc transporter Zrt1 (3). Recent studies have shown that acquisition of the macronutrient phosphate (P_i_) is also essential for C. albicans survival in host environments (4, 5). This is perhaps unsurprising, as P_i_ is an essential component of nucleic acids and phospholipids and, in the form of ATP, is a universal cellular energy source. Moreover, due to its negative charge at physiological pH, P_i_ is extensively used as a signaling molecule via the posttranslational modification of numerous proteins (6).

The systems involved in P_i_ acquisition have been extensively studied in the model yeast Saccharomyces cerevisiae. Following growth under P_i_-limiting conditions, S. cerevisiae activates the PHO pathway, which culminates in the nuclear accumulation of the Pho4 transcription factor and the induction of a suite of genes involved in P_i_ acquisition and storage (7). This pathway is seemingly conserved in C. albicans, as P_i_ starvation similarly elicits the nuclear accumulation of a Pho4 orthologue and the Pho4-dependent expression of genes involved in P_i_ homeostasis (5). Proteins involved in P_i_ acquisition include secreted acid phosphatases such as Pho100 and the high-affinity P_i_ transporter Pho84, which collectively facilitate scavenging of P_i_ from the extracellular environment. Consequently, C. albicans cells lacking Pho4 struggle to grow in P_i_-limiting environments and under alkaline pH conditions, as this also triggers a P_i_ starvation response (5, 8). Notably, Pho4, Pho100, and Pho84 have all been shown to contribute to C. albicans pathogenesis, illustrating that P_i_ acquisition is an important virulence trait in this fungal pathogen (5, 9, 10).

Despite the essential role of P_i_, cytosolic levels of P_i_ are tightly controlled, as cells need to balance the biosynthetic and signaling requirements for P_i_ against elevated cytosolic P_i_ levels. This is because, as a product of all nucleotide-hydrolyzing reactions, P_i_ has the potential to stall metabolism if levels get too high (11). Thus, the PHO pathway also regulates genes involved in P_i_ storage, including components of the vacuole transporter chaperone (VTC) complex, which synthesizes the P_i_ storage molecule polyphosphate (polyP). Synthesis and translocation of polyP into the vacuole are tightly linked, possibly because polyP produced in the cytosol is toxic and this mechanism ensures buffering of cytosolic P_i_ levels (12). Indeed, the majority of polyP in fungal cells is stored in the vacuole, with only small pools located in the cytoplasm, mitochondria, and nucleus (13). The S. cerevisiae VTC complex is a heterotrimer composed of three structural units (Vtc1, Vtc2, and Vtc3) and the polyP synthetase Vtc4 (14). A fifth subunit, Vtc5, has recently been shown to physically associate with VTC complex to accelerate polyP synthesis (15). The VTC complex, located on the vacuolar membrane, synthesizes linear polymers ranging from 10 to several hundred P_i_ molecules linked by high-energy phosphoanhydride bonds (16). Release of P_i_ from polyP stores is regulated by several polyphosphatases, including Ppn1 and Ppx1 (reviewed in reference 17). Ppn1 is a vacuolar endopolyphosphatase that cleaves internal phosphoanhydride bonds (18) and depends on vacuolar proteases for activation (19). In contrast, Ppx1 is a cytoplasmic enzyme and exhibits potent exopolyphosphatase activity, releasing P_i_ from the ends of polyP chains (20), although it can also function as an endophosphatase under certain conditions (21). *PPN1*, but not *PPX1*, is induced in response to P_i_ starvation in S. cerevisiae (22). The same holds true in C. albicans, where Pho4 regulates the induction of *PPN1* following P_i_ limitation (5). Intriguingly, it is not yet known how the opposing functions of polyP synthesis and mobilization are coordinated to maintain optimal cellular P_i_ levels.

PolyP is one of the most ancient and conserved molecules in biology, and although it was once dismissed as a “molecular fossil,” there has been a resurgence of interest in this enigmatic polymer due to its emergence as more than simply a P_i_ storage molecule (16). The pioneering work of Arthur Kornberg and colleagues revealed a number of diverse functions for polyP in bacteria, including, stress resistance, motility, quorum sensing, biofilm formation, and virulence (reviewed in reference 23). More recently, polyP has been shown to function as a potent chaperone that protects bacterial cells against stress-induced protein aggregation, including oxidative stress (24). Less is known about polyP function in eukaryotes, although recent studies in S. cerevisiae have uncovered a role for polyP in cell cycle progression and genome stability (25), and there is a significant body of work linking polyP with osmoregulation in trypanosomes (26) and blood coagulation in humans (27). Furthermore, the finding that in eukaryotes polyP can be added to proteins has revealed polyphosphorylation to be a novel regulatory posttranslational protein modification (28).

Notably, disruption of P_i_ homeostasis in C. albicans results in a myriad of phenotypes in addition to impaired growth under P_i_-limiting conditions (5, 9, 29, 30). For example, loss of Pho4 results in cells that are exquisitely sensitive to superoxide stress and cationic stresses, and similar phenotypes are seen upon loss of the P_i_ transporter Pho84 (5, 9). As polyP has been implicated in stress resistance in several organisms, it was reasoned that the stress-sensitive phenotypes attributed to loss of Pho4 could be due to the lack of polyP in these cells. However, extensive phenotypic analysis of C. albicans mutants (*vtc1*Δ and *vtc4*Δ mutants) lacking polyP revealed few stress-protective roles for this polymer (5). Indeed, the only function attributed to the presence of polyP thus far is as a manganese storage reservoir. However, polyP is rapidly mobilized in response to a number of stresses in addition to P_i_ limitation in C. albicans (5). This suggests that the ability to mobilize P_i_ from polyP may be important for P_i_ homeostasis and stress resistance. Here, we set out to explore the cellular processes that require polyP mobilization in C. albicans and its importance in virulence.

## RESULTS

### The polyphosphatases Ppn1 and Ppx1 exhibit functional redundancy.

**FIG 1:**
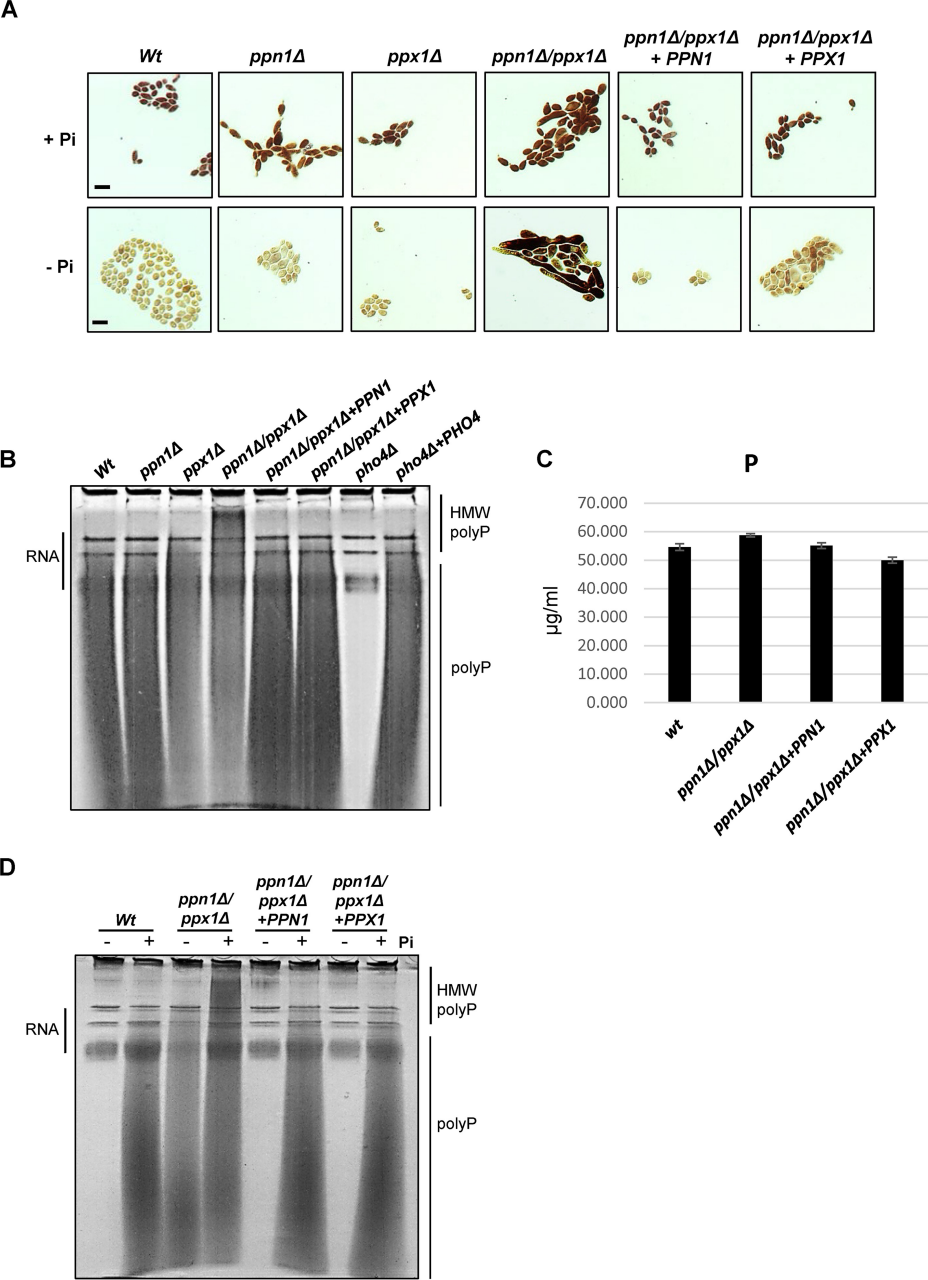
Ppn1 and Ppx1 function redundantly to mobilize polyP stores. (A) Neisser staining of the indicated strains following growth in YPD+P_i_ or YPD-LPi for 16 h. Bars represent 10 μm, and the scale is the same across each row of images. (B) Toluidine blue staining of RNA/polyP extracts after electrophoresis on urea-polyacrylamide gels from cells grown in YPD. The high-molecular-weight (HMW) polyP in *ppn1*Δ *ppx1*Δ cells is indicated. (C) Impact of *PPX1* and *PPN1* loss on intracellular phosphate levels. Whole-cell nitric acid digests of WT, *ppn1*Δ *ppx1*Δ, *ppn1*Δ *ppx1*Δ+*PPN1*, and *ppn1*Δ *ppx1*Δ+*PPX1* cells grown in YPD were analyzed by ICP-MS. Phosphate levels shown are means and SD for three independent cultures. (D) Toluidine blue staining of RNA/polyP extracts after electrophoresis on urea-polyacrylamide gels from cells grown in YPD+P_i_ or YPD-LPi for 16 h.

To identify polyphosphatases in C. albicans we used the amino acid sequences of the S. cerevisiae Ppx1 exopolyphosphatase and the Ppn1 endo/exopolyphosphatase to conduct a BLAST search against the C. albicans genome database. This identified both Ppx1 (C2_06110W_A) and Ppn1 (C7_00980W_A) homologues in C. albicans with 34% and 44% identity to the respective S. cerevisiae proteins (see Fig. S1 in the supplemental material). To explore functional conservation, we generated C. albicans strains lacking *PPX1* and *PPN1* homologues and examined polyP levels and the size of polyP chains in these mutants. PolyP can be detected in whole cells by Neisser staining (31) and in cell extracts by toluidine blue staining of polyP resolved on polyacrylamide gels (32). Interestingly, deletion of either *PPN1* or *PPX1* had no obvious impact on polyP levels (Fig. 1A) or on the size of the polyP chains (Fig. 1B). Hence, to explore potential functional redundancy between Ppn1 and Ppx1 in C. albicans, a double *ppx1*Δ *ppn1*Δ mutant was created. Increased Neisser staining of cells indicated higher levels of polyP in the *ppx1*Δ *ppn1*Δ mutant compared to wild-type cells (Fig. 1A), and resolution of polyP by PAGE revealed the presence of longer polyP chains upon deletion of both polyphosphatases (Fig. 1B). Despite the presence of longer polyP chains in the *ppx1*Δ *ppn1*Δ mutant, no significant differences in overall cellular P_i_ levels compared to wild-type cells were evident (Fig. 1C). However, reintegration of either *PPN1* or *PPX1* reduced the levels and size of polyP to those seen in wild-type cells (Fig. 1B). These results indicate that Ppx1 and Ppn1 have redundant functions in releasing P_i_ from polyP in C. albicans.

10.1128/mbio.00342-22.1 FIG S1 Sequence alignment of Ppn1 and Ppx1 orthologues in S. cerevisiae and C. albicans. Sequences were aligned using ClustalW. Identical amino acids are annotated with asterisks, conserved amino acids with colons, and semiconserved amino acids with dots. Gaps introduced to maximize alignment are indicated with dashes. (A) Sequences of C. albicans (Ca) Ppn1 and S. cerevisiae (Sc) Ppn1. The endopolyphosphatase domain is highlighted in yellow. (B) Sequences of C. albicans (Ca) Ppx1 and S. cerevisiae (Sc) Ppx1. The Ppx1 superfamily domain is highlighted in red and the DHHA2 domain in yellow. Download FIG S1, TIF file, 1.9 MB. Copyright © 2022 Ahmed et al. 2022 Ahmed et al. https://creativecommons.org/licenses/by/4.0/ This content is distributed under the terms of the Creative Commons Attribution 4.0 International license.

We next asked whether Ppn1 and Ppx1 are required for the active liberation of P_i_ from polyP in response to P_i_ starvation. Consistent with previous results (5), polyP stores are completely mobilized in wild-type cells following 16 h P_i_ starvation (Fig. 1A and D). In contrast, polyP mobilization is drastically impaired in *ppn1*Δ *ppx1*Δ cells, as Neisser staining revealed similar levels of polyP in this mutant irrespective of the level of P_i_ in the growth media (Fig. 1A). Such analysis also indicated that *ppn1*Δ *ppx1*Δ cells exhibit morphological defects, which is examined in more detail below. Following resolution and visualization of polyP on polyacrylamide gels, it was evident that some of the higher-molecular-weight polyP chains, seen in *ppn1*Δ *ppx1*Δ cells, are resolved to shorter chains following P_i_ starvation (Fig. 1D). Notably, reintegration of either *PPX1* or *PPN1* into *ppn1*Δ *ppx1*Δ cells completely restored the ability of cells to mobilize polyP following P_i_ starvation (Fig. 1D). Collectively, these results indicate that Ppn1 and Ppx1 function redundantly in C. albicans to mobilize polyP under P_i_-limiting conditions and that both polyphosphatases need to be inactivated before longer polyP chains accumulate under P_i_-replete conditions.

### Prevention of polyP mobilization impacts the activation of the PHO pathway.

**FIG 2:**
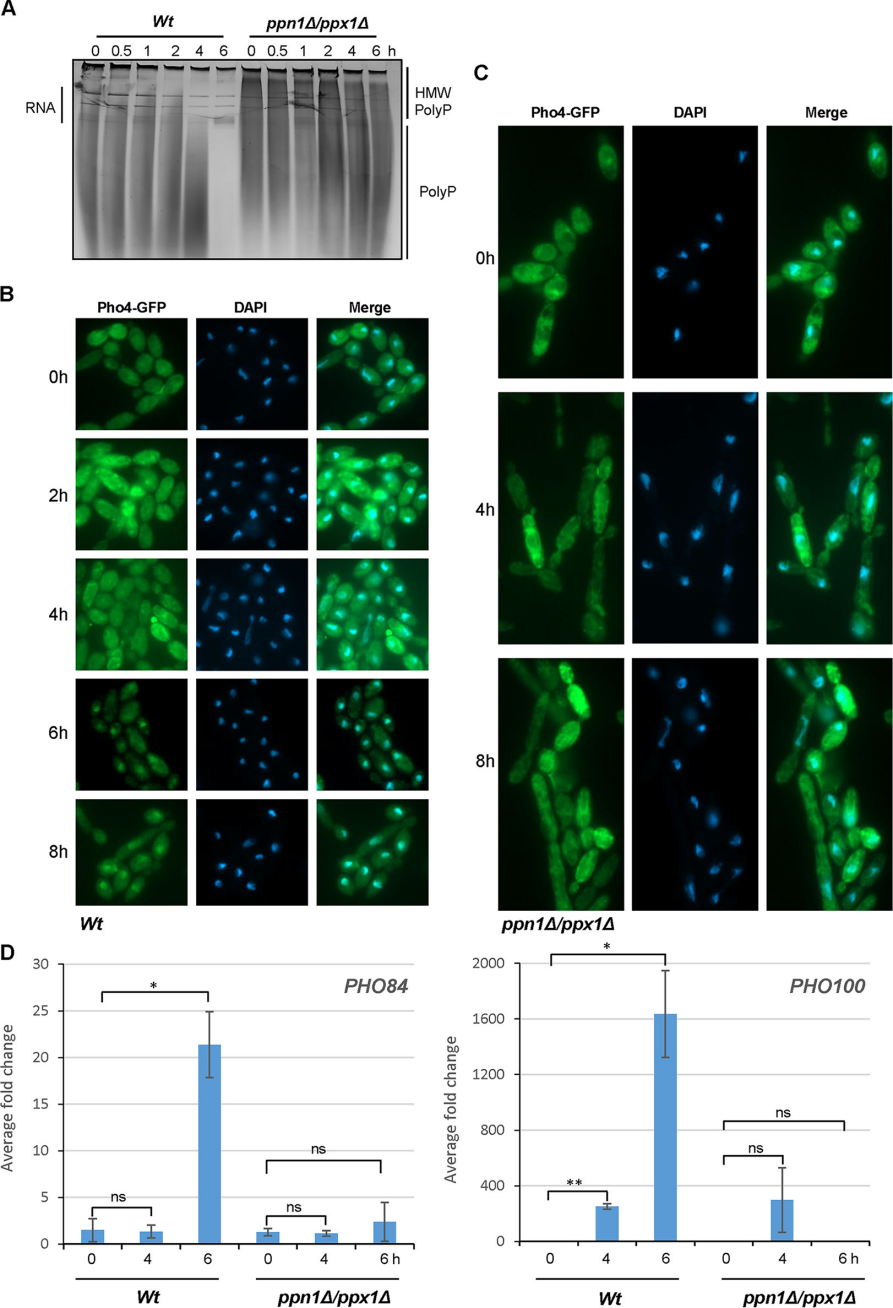
Pho4 activation in *ppn1*Δ *ppx1*Δ cells. (A) Toluidine blue staining of RNA/polyP extracts after electrophoresis on urea-polyacrylamide gels from WT and *ppn1*Δ *ppx1*Δ cells expressing Pho4-GFP grown in YPD until mid-log phase (*t* = 0) and then moved to YPD-LPi medium for the indicated times. (B and C) Cells from the cultures described above were processed to examine Pho4-GFP localization using fluorescence microscopy. DAPI staining illustrates nuclear positioning. (D) RT-qPCR analysis showing fold induction of the Pho4 target genes *PHO84* and *PHO100* after growth for 4 and 6 h in YPD-LPi. Transcript levels were measured relative to the internal *ACT1* mRNA control and normalized to the level of transcript in WT cells with P_i_. Means and standard deviations for three biological replicates are shown. ns, not significant; *, *P* < 0.05; **, *P* < 0.01.

As cells respond to P_i_ limitation by mobilizing P_i_ stores from polyP, we asked whether preventing polyP mobilization would have an impact on activation of the PHO pathway, such as preventing Pho4 nuclear accumulation and the induction of Pho4-dependent genes. First, the kinetics of polyP mobilization and Pho4 nuclear accumulation were examined. Wild-type and *ppn1*Δ *ppx1*Δ cells expressing Pho4-GFP were grown under both P_i_-replete and P_i_-limiting conditions, and cell samples were dually processed to examine polyP levels and the cellular localization of Pho4. PolyP mobilization was visualized by Neisser staining of polyP following urea-PAGE (Fig. 2A) and fluorescence microscopy was employed to determine the cellular localization of Pho4 (Fig. 2B and 2C). In wild-type cells, some polyP mobilization was evident after 4 h growth in P_i_-limiting medium, with complete mobilization occurring by 6 h (Fig. 2A). Such mobilization was severely impaired in *ppn1*Δ *ppx1*Δ cells (Fig. 2A), consistent with that observed previously (Fig. 1D). Examining the cellular localization of Pho4 revealed that nuclear accumulation of Pho4 was seen only in wild-type cells following 6 h growth in P_i_-limiting medium (Fig. 2B). In contrast, nuclear accumulation of Pho4 was not observed in *ppn1*Δ *ppx1*Δ cells following 8 h (Fig. 2C) or even 16 h (Fig. S2A) growth in P_i_-limiting medium. In some *ppn1*Δ *ppx1*Δ cells, a punctate staining pattern was seen, but this did not colocalize with the DAPI (4′,6-diamidino-2-phenylindole) nuclear stain (Fig. 2C). This deregulation of localization is not due to processing of the Pho4-GFP fusion, as Western blotting using a green fluorescent protein (GFP) antibody revealed that Pho4-GFP is intact in *ppn1*Δ *ppx1*Δ cells (Fig. S2B). These results, showing that Pho4 nuclear accumulation (i) coincides with the complete mobilization of polyP and (ii) is inhibited when polyP mobilization is impaired, suggest that polyP mobilization precedes Pho4 activation in C. albicans.

10.1128/mbio.00342-22.2 FIG S2 Analysis of PHO pathway activation after extended growth in P_i_-limiting medium. (A) WT and *ppn1*Δ *ppx1*Δ cells expressing Pho4-GFP were grown in YPD until mid-log phase (*t* = 0), moved to YPD-LPi medium for 16 h, and processed to examine Pho4-GFP localization using fluorescence microscopy. DAPI staining illustrates nuclear positioning. (B) WT and *ppn1*Δ *ppx1*Δ cells expressing Pho4-GFP were grown in YPD-LPi medium for 16 h, and Pho4-GFP expression was examined by Western blotting of whole-cell extracts using an anti-GFP antibody. An antitubulin antibody was used as a loading control. (C) RT-qPCR analysis showing fold induction of Pho4 target genes *PHO84* and *PHO100* after growth of the indicated strains for 16 h in YPD-LPi. Transcript levels were measured relative to the internal *ACT1* mRNA control and normalized to the level of transcript in WT cells with P_i_. Means and standard deviations for three biological replicates are shown. ns, not significant; *, *P* < 0.05; **, *P* < 0.01. Download FIG S2, TIF file, 2.7 MB. Copyright © 2022 Ahmed et al. 2022 Ahmed et al. https://creativecommons.org/licenses/by/4.0/ This content is distributed under the terms of the Creative Commons Attribution 4.0 International license.

As nuclear accumulation of Pho4 following P_i_ limitation was not evident in *ppn1*Δ *ppx1*Δ cells, we investigated whether this relayed to changes in the transcript profile of Pho4-dependent genes, including the high-affinity P_i_ symporter *PHO84* and the secreted acid phosphatase *PHO100.* Both of these genes are induced in a Pho4-dependent manner in response to P_i_ starvation in C. albicans to facilitate P_i_ acquisition from external sources (5). Reverse transcription-quantitative PCR (RT-qPCR) revealed that both *PHO84* and *PHO100* were highly expressed in wild-type cells following 6 h growth under P_i_-limiting conditions, with some induction of *PHO100* also observed after 4 h (Fig. 2D). However, such induction was drastically reduced in *ppn1*Δ *ppx1*Δ cells (Fig. 2D), even following extended growth in P_i_-limiting conditions (Fig. S2C), which is consistent with the lack of Pho4 nuclear accumulation. This impaired induction of P_i_ acquisition genes is not due to significantly slower growth of *ppn1*Δ *ppx1*Δ cells (Fig. S3A). Furthermore, reintegration of either *PPN1* or *PPX1* into *ppn1*Δ *ppx1*Δ cells fully restored *PHO100* induction and partially restored *PHO84* induction (Fig. S3B). To explore the links between polyP and Pho4 activation further, the induction of *PHO100* and *PHO84* in *vtc4*Δ cells lacking polyP was also determined. In contrast to that seen in *ppn1*Δ *ppx1*Δ cells, induction of these genes was not impaired in *vtc4*Δ cells, with faster activation of *PHO100* occurring in cells lacking polyP (Fig. S3C). Taken together, these results show that polyP mobilization coincides with PHO pathway activation in C. albicans and that inhibiting P_i_ release from polyP via deletion of Ppn1 and Ppx1 impairs both the nuclear accumulation of Pho4 and the induction of Pho4-dependent genes.

10.1128/mbio.00342-22.3 FIG S3 (A) Analysis of growth of the indicated strains under the conditions used for cells in the RT-qPCR experiments. Cells numbers rather than OD were recorded due to morphological defects of *ppn1*Δ *ppx1*Δ cells. Means and standard deviations for three biological replicates are shown. (B) Reintegration of *PPN1* or *PPX1* restores Pho4-dependent gene expression. RT-qPCR analysis of Pho4 target genes *PHO84* and *PHO100* in the indicated strains. Means and standard deviations for three biological replicates are shown. ns, not significant; *, *P* < 0.05; **, *P* < 0.01. (C) Pho4-dependent gene expression occurs with faster kinetics in cells lacking polyP. RT-qPCR analysis of Pho4 target genes *PHO84* and *PHO100* in wild-type and *vtc4*Δ strains. Means and standard deviations for three biological replicates are shown. ns, not significant; *, *P* < 0.05; **, *P* < 0.01. Download FIG S3, TIF file, 1.6 MB. Copyright © 2022 Ahmed et al. 2022 Ahmed et al. https://creativecommons.org/licenses/by/4.0/ This content is distributed under the terms of the Creative Commons Attribution 4.0 International license.

### PolyP mobilization contributes to stress resistance.

**FIG 3:**
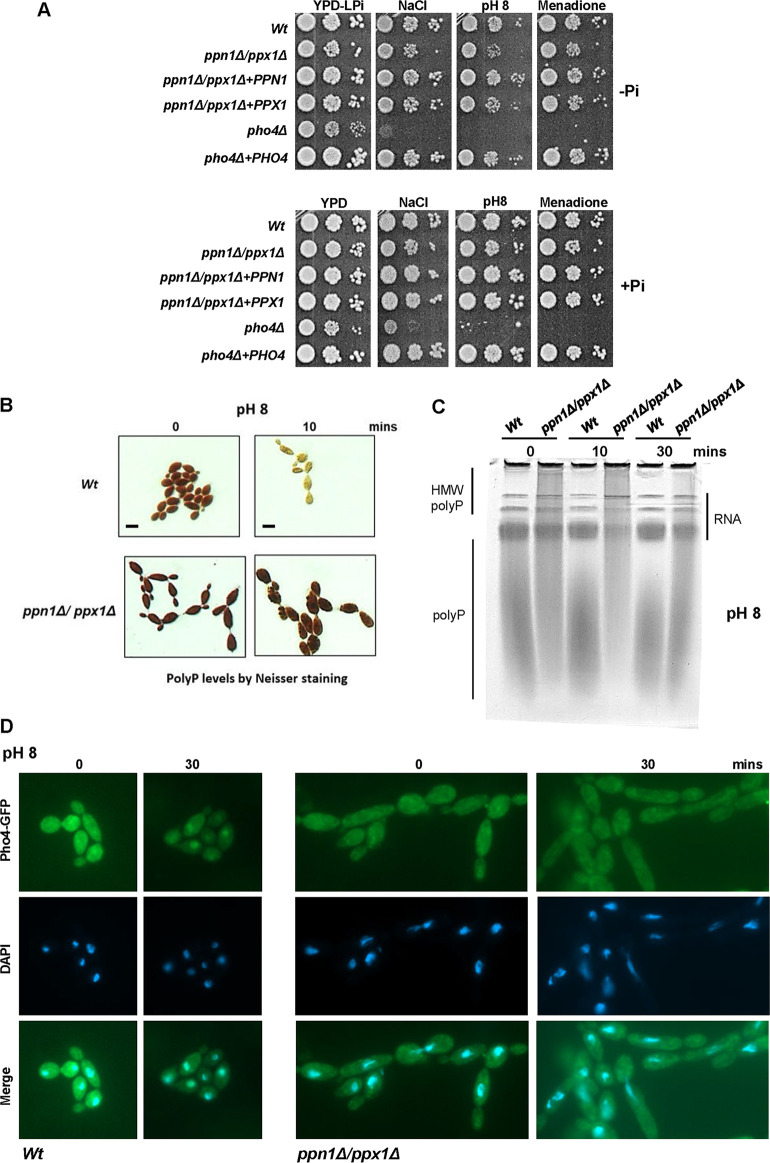
PolyP mobilization and stress responses. (A) PolyP mobilization promotes stress resistance under P_i_-limiting conditions. Exponentially growing strains were spotted in serial dilutions onto YPD-LPi plates containing 1 M NaCl and 300 μM menadione or at pH 8 (top, −P_i_) or in YPD plates containing the same stresses (bottom, +P_i_). Plates were incubated for 24 h at 30°C. (B) PolyP mobilization in response to alkaline stress is dependent on Ppn1 and Ppx1. Neisser staining of the indicated strains grown in YPD or after 10 min growth in YPD (pH 8). Bar, 10 μm. (C) Toluidine blue staining of RNA/polyP extracts from indicated strains after electrophoresis on urea-polyacrylamide gels, before and after 30 min growth in YPD medium (pH 8). (D) WT and *ppn1*Δ *ppx1*Δ cells expressing Pho4-GFP were grown in YPD, left untreated or transferred to YPD medium (pH 8) for 30 min, and processed to examine Pho4-GFP localization using fluorescence microscopy. DAPI staining illustrates nuclear positioning.

Previously we reported that cells lacking the Pho4 transcription factor display significantly impaired resistance to alkaline stress, cationic stress, and menadione-imposed superoxide stress (5). However, analysis of the Pho4-mediated transcriptome indicated that this transcription factor does not directly regulate stress-protective genes (5). Thus, an alternative possibility is that reduced intracellular P_i_ levels in *pho4*Δ cells may contribute to the stress-sensitive phenotypes of this mutant. Consistent with this is the observation that P_i_ is mobilized from polyP stores following exposure to either cationic or alkaline stress (5). Therefore, we asked whether preventing polyP mobilization impacts resistance to stresses known to require Pho4 for resistance. Spot test assays were performed on P_i_-limiting medium with the expectation that polyP mobilization would be more important under such conditions. Figure 3 illustrates that *ppn1*Δ *ppx1*Δ cells do display some impaired resistance to alkaline, cationic, and superoxide stress when grown under P_i_-limiting conditions, albeit not to the same levels as *pho4*Δ cells (Fig. 3A). However, stress-sensitive phenotypes were not evident on YPD medium containing P_i_ (Fig. 3A) or with the single *ppn1*Δ and *ppx1*Δ mutants (Fig. S4). This indicates that Ppn1 and Ppx1 contribute to stress resistance only under P_i_-limiting conditions and, as seen before, that these polyphosphatases function redundantly to promote stress resistance. Consistent with this, the impaired stress resistance associated with Ppn1 and Ppx1 loss was rescued upon reintegration of either *PPN1* or *PPX1* (Fig. 3A). These results show that polyP mobilization does contribute to the cellular resistance to stresses that are also dependent on the Pho4 transcription factor.

10.1128/mbio.00342-22.4 FIG S4 Stress phenotypes of the single Ppn1 and Ppx1 mutants. Exponentially growing strains were spotted in serial dilutions onto YPD-LPi plates containing the indicated stresses. Download FIG S4, TIF file, 1 MB. Copyright © 2022 Ahmed et al. 2022 Ahmed et al. https://creativecommons.org/licenses/by/4.0/ This content is distributed under the terms of the Creative Commons Attribution 4.0 International license.

It has been documented in S. cerevisiae that alkaline stress triggers a P_i_ starvation response (8) which is accompanied by a rapid mobilization of polyP (33). Consistent with this, alkaline stress in C. albicans triggers polyP mobilization and the subsequent nuclear accumulation of Pho4 (5). As *ppx1*Δ *ppn1*Δ cells display some sensitivity to alkaline stress (Fig. 3A), we examined whether polyP mobilization and Pho4 nuclear accumulation, following growth in pH 8 medium, were dependent on Ppn1 and Ppx1. Both Neisser staining of cells and visualization of polyP by urea-PAGE replicated previous findings that polyP is rapidly mobilized in wild-type cells following alkaline stress (Fig. 3B and C). However, this alkaline stress-stimulated P_i_ mobilization was significantly impaired in *ppn1*Δ *ppx1*Δ cells. In wild-type cells, mobilization of polyP was evident after 10 min after switching to pH 8 medium, but this was not seen in cells lacking Ppn1 and Ppx1. Mobilization of polyP is also dependent on Ppx1 and Ppn1 when cells are grown in medium at pH 7.4 (Fig. S5), which reflects the slightly alkaline pH of blood. As impaired polyP mobilization impacted the nuclear accumulation of Pho4 following P_i_ limitation (Fig. 2C), we next examined the cellular localization of Pho-GFP in wild-type and *ppn1*Δ *ppx1*Δ cells following alkaline pH stress. As shown in Fig. 3D, Pho4 clearly accumulated in the nucleus in wild-type cells following growth in pH 8 medium for 30 min. In contrast, no nuclear accumulation of Pho4 was evident in *ppn1*Δ *ppx1*Δ cells following the same treatment. Collectively, these results indicate that polyP mobilization, mediated by Ppn1 and Ppx1, plays an important role in the cellular response of C. albicans to alkaline stress.

10.1128/mbio.00342-22.5 FIG S5 PolyP mobilization in response to growth at pH 7.4 is dependent on Ppn1 and Ppx1. Neisser staining of the indicated strains grown in YPD and after 60 min growth in YPD (pH 7.4). Download FIG S5, TIF file, 2.3 MB. Copyright © 2022 Ahmed et al. 2022 Ahmed et al. https://creativecommons.org/licenses/by/4.0/ This content is distributed under the terms of the Creative Commons Attribution 4.0 International license.

### Cells lacking both polyphosphatases display severe morphological defects.

**FIG 4:**
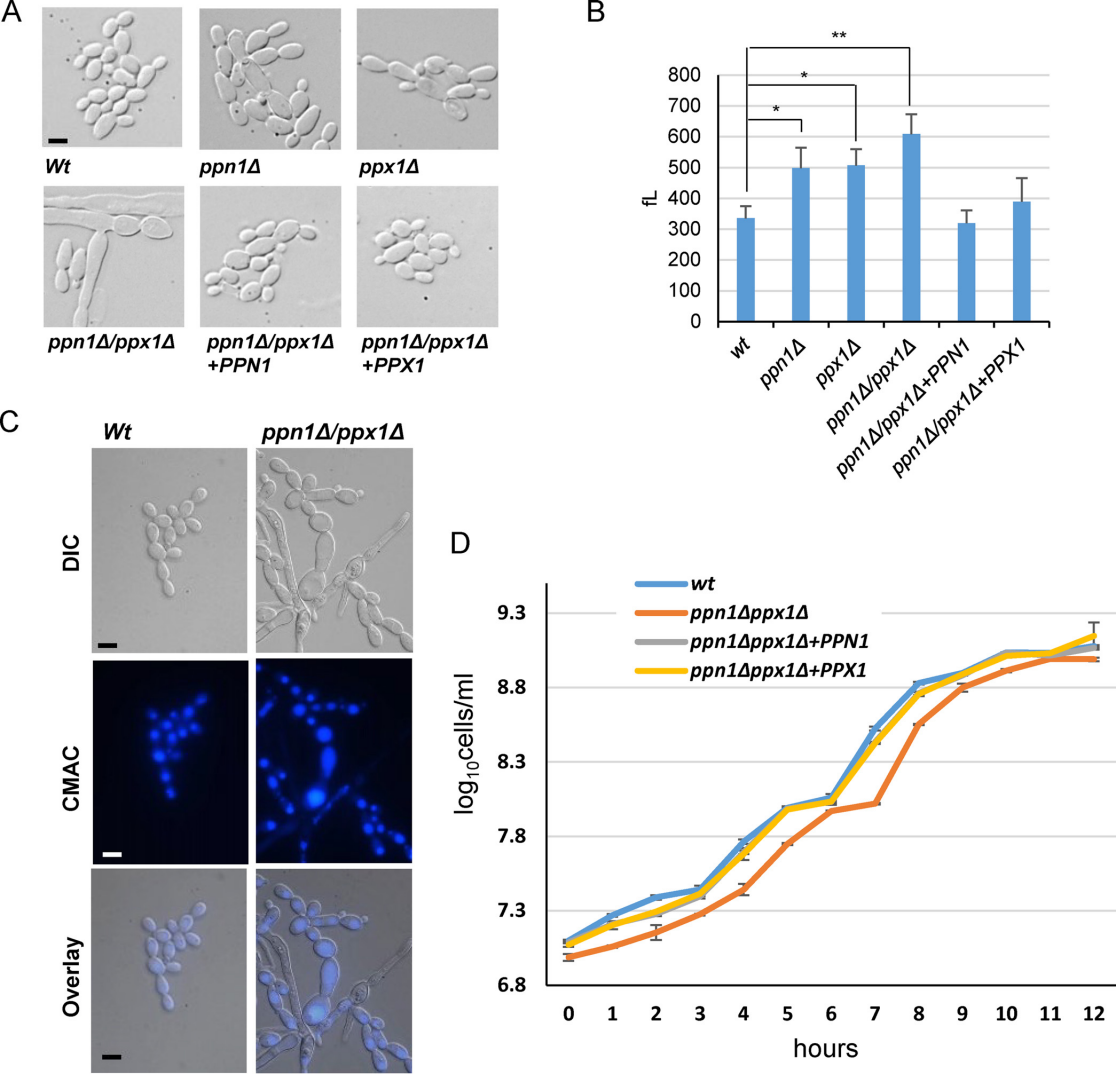
Morphological and growth characteristics of *ppn1*Δ *ppx1*Δ cells. (A) Cells lacking Ppn1 and Ppx1 have altered morphologies. DIC images of exponentially growing cells. (B) Cells lacking Ppn1 and Ppx1 are larger than wild-type cells. Cell volume analysis showing the cell volumes (means and SD). The data were analyzed statistically using Student’s two-sample *t* test. ns, not significant; *, *P* < 0.05; **, *P* < 0.01. (C) Some *ppn1*Δ *ppx1*Δ cells exhibit large vacuoles. Vacuolar morphology was captured by CMAC staining. (D) Cells lacking *PPN1* and *PPX1* have a slight slow-growth phenotype. Analysis of growth of the indicated strains in YPD. Cells numbers rather than OD were recorded due to the morphological defects seen in *ppn1*Δ *ppx1*Δ cells. Bars (A and C) represent 10 μm, and scale is the same across each row of images.

During our investigations into polyP mobilization in C. albicans, we observed morphological defects that were particularly evident in cells lacking both Ppn1 and Ppx1 (Fig. 4A). Cell volume analysis revealed that loss of *PPN1* or *PPX1* led to a significant increase in cell volume, which was exacerbated in the double *ppn1*Δ *ppx1*Δ mutant and reversed upon reintegration of *PPN1* or *PPX1* (Fig. 4B). The larger cell size can also be seen in the Neisser-stained cells in Fig. 1A and the fluorescence microscopy images in Fig. 2C and 3D. In addition to larger yeast cells, *ppn1*Δ *ppx1*Δ cells often formed swollen pseudohypha-like structures under non-filament-inducing conditions (Fig. 4A). As the majority of polyP is stored in the vacuole, we asked whether prevention of polyP mobilization would result in altered vacuolar morphology. Microscopy of cells stained with the vacuolar marker dye 7-amino-4-chloromethylcoumarin (CMAC) revealed that a proportion of *ppn1*Δ *ppx1*Δ cells have highly expanded vacuoles. Moreover, such expanded vacuoles tended to be restricted to cells displaying highly abnormal morphologies (Fig. 4C). Vacuole volume is implicated in cell cycle control in C. albicans (34), and thus, such expanded vacuoles seen in some *ppn1*Δ *ppx1*Δ cells may contribute to their morphological defects. Indeed, blocking polyP mobilization results in cells that grow slightly more slowly than wild-type cells (Fig. 4D); the doubling time for wild-type cells was 64.3 ± 0.6 min, and that for *ppn1*Δ *ppx1*Δ cells was 70.7 ± 1.2 min (*P = *0.001).

**FIG 5:**
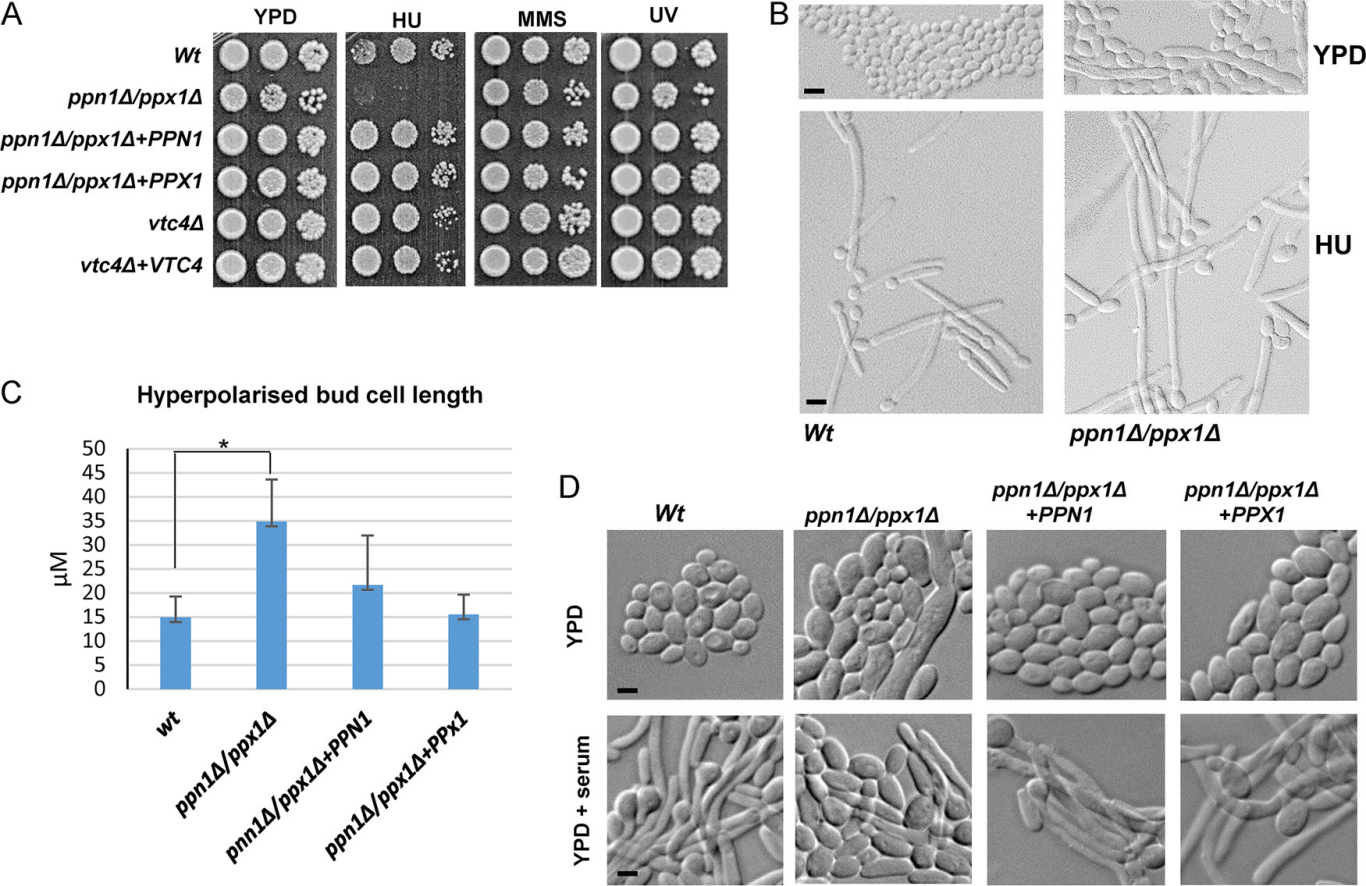
Ppn1 and Ppx1 are required for replication stress resistance and the formation of hyphae. (A) Exponentially growing strains were spotted in serial dilutions onto YPD plates that contained HU (40 mM) or MMS (0.02%) or that were exposed to UV (75 J/m^2^). Plates were incubated for 24 h at 30°C. (B) DIC images of cells grown in YPD and following treatment with 40 mM HU for 4 h. Bars represent 10 μm, and scale is the same across each row of images. (C) Quantification of hyperpolarized bud length was carried out using Zeiss imaging software on 200 cells for each strain. Data are means and SD. Statistical analysis was performed using Student’s two-sample *t* test. *, *P* < 0.05. (D) Stationary-phase cells were diluted 1:10 in YPD medium containing 10% fetal bovine serum and incubated at 37°C for 3 h (YPD + serum). Bars represent 10 μm, and scale is the same across each row of images.

Recent work in S. cerevisiae has shown that polyP levels show a cyclical decrease during the S phase of the cell cycle. Furthermore, loss of Ppn1 and Ppx1 leads to a delay in progression through the G_1_/S phase of the cell cycle which correlates with impaired deoxyribonucleoside triphosphate (dNTP) production (25). These findings support a model whereby P_i_ release from polyP is important to support the synthesis of dNTPs necessary for DNA replication. Due to the morphological defects exhibited by C. albicans *ppn1*Δ *ppx1*Δ cells, we were unable to obtain a synchronous population (via centrifugal elutriation) to ask whether such cells also exhibited an S phase delay. Hence, instead, we asked whether such cells were sensitive to DNA replication stress elicited by hydroxyurea (HU), a potent inhibitor of ribonucleotide reductase (RNR) which synthesizes the dNTPs necessary for replication (35). Cells lacking both polyphosphatases were extremely sensitive to HU, and this sensitivity was rescued by reintegration of either *PPN1* or *PPX1* (Fig. 5A). Moreover, such sensitivity was restricted to HU-mediated replication stress and was not seen with other genotoxic stresses such as UV light or the DNA alkylating agent methyl methanesulfonate (MMS). Next, we explored whether cells lacking polyP would also show sensitivity to DNA stress, but as illustrated in Fig. 5A, cells lacking the polyP polymerase Vtc4 displayed wild-type levels of resistance to HU. This suggests that an inability to mobilize, rather than synthesize, polyP results in cells acutely sensitive to replication stress. Relevant to this, an interesting phenomenon has been reported in C. albicans, in which a range of genotoxic stresses, including replication stress, induce filamentous growth due to the significant elongation of the daughter bud (36, 37). Thus, we examined whether HU-induced hyperpolarized bud growth was impacted in *ppn1*Δ *ppx1*Δ cells. Consistent with previous results (36, 37), wild-type cells formed extensive filaments following HU exposure (Fig. 5B). However, *ppn1*Δ *ppx1*Δ cells demonstrated a heightened response in that longer filaments were generated following HU exposure than in wild-type cells (Fig. 5B). Quantification of filament length revealed statistically different differences between those formed in wild-type and *ppn1*Δ *ppx1*Δ cells and showed that reintegration of either PPN1 or PPX1 reversed this difference (Fig. 5C). The greater extent of filamentation seen in *ppn1*Δ *ppx1*Δ cells may be linked to the enhanced sensitivity of such cells to HU, due to delays in S phase progression.

Morphological switching is a key virulence determinant in C. albicans (38). Therefore, we examined whether the morphological defects presented by *ppn1*Δ *ppx1*Δ cells impacted the ability of this strain to undergo morphological switching to form true hyphae. As expected, wild-type cells rapidly formed true hyphae when grown under the filament-inducing conditions of medium supplemented with 10% serum at 37°C (Fig. 5D). Strikingly, serum-induced filamentation was drastically impaired in the *ppn1*Δ *ppx1*Δ mutant, with only very few cells forming true hypha-like structures (Fig. 5D). This defect was completely reversed upon reintegration of either *PPN1* or *PPX1*, which indicates that preventing polyP mobilization inhibits morphological switching in C. albicans, a key virulence trait in this fungal pathogen.

### PolyP mobilization is required for virulence in multiple infection models.

**FIG 6:**
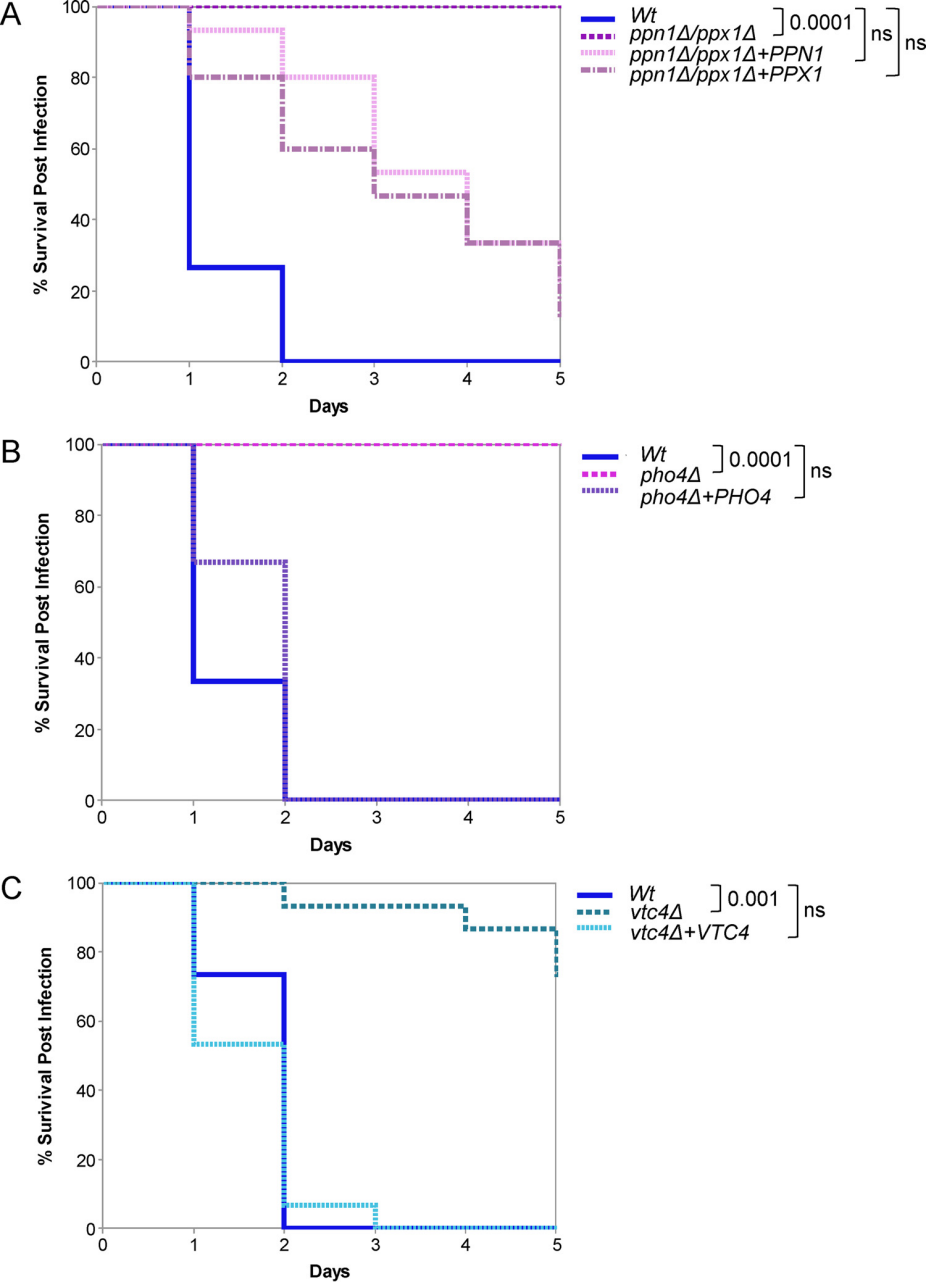
Virulence analysis in the Galleria mellonella model of infection. (A to C) Comparison of virulence of the indicated strains in the *Galleria* model of systemic infection (15 larvae per fungal strain). The data were analyzed statistically using the log-rank (Mantel-Cox) test. ns, not significant.

Given that preventing polyP mobilization impacts a number of virulence traits in C. albicans, including stress resistance and morphological switching, we investigated the importance of polyP mobilization in C. albicans virulence using the Galleria mellonella invertebrate model of systemic candidiasis (39). Cells lacking both *PPX1* and *PPN1* displayed significantly attenuated virulence compared to wild-type cells (*P* < 0.001) (Fig. 6A). Interestingly, despite the redundancy displayed by Ppn1 and Ppx1 in previous experiments, reconstitution with either *PPN1* or *PPX1* only partially restored infectivity to that of wild-type cells (Fig. 6A).

As polyP mobilization is important for the activation of Pho4, we predicted that Pho4 would also be an important virulence determinant in the G. mellonella model. As shown in Fig. 6B, *pho4*Δ cells display significantly impaired virulence in this model of infection (*P* < 0.001), similar to that exhibited by *ppn1*Δ *ppx1*Δ cells. Thus, P_i_ mobilization and acquisition are important for the virulence of C. albicans in the G. mellonella model, which is consistent with our findings that polyP mobilization is an essential prerequisite for Pho4-mediated P_i_ acquisition.

To explore the importance of polyP presence in mediating C. albicans virulence, *vtc4*Δ cells were also tested in the G. mellonella model. Interestingly, deletion of *VTC4* did impair C. albicans virulence (*P* < 0.01), albeit not to the same extent as that observed for *pho4*Δ and *ppn1*Δ *ppx1*Δ cells (Fig. 6C). Thus, although *in vitro* experiments have yet to reveal the precise roles of polyP in C. albicans biology (with the exception of manganese storage [5]), the presence of polyP does appear to contribute to the virulence of this important human fungal pathogen.

**FIG 7:**
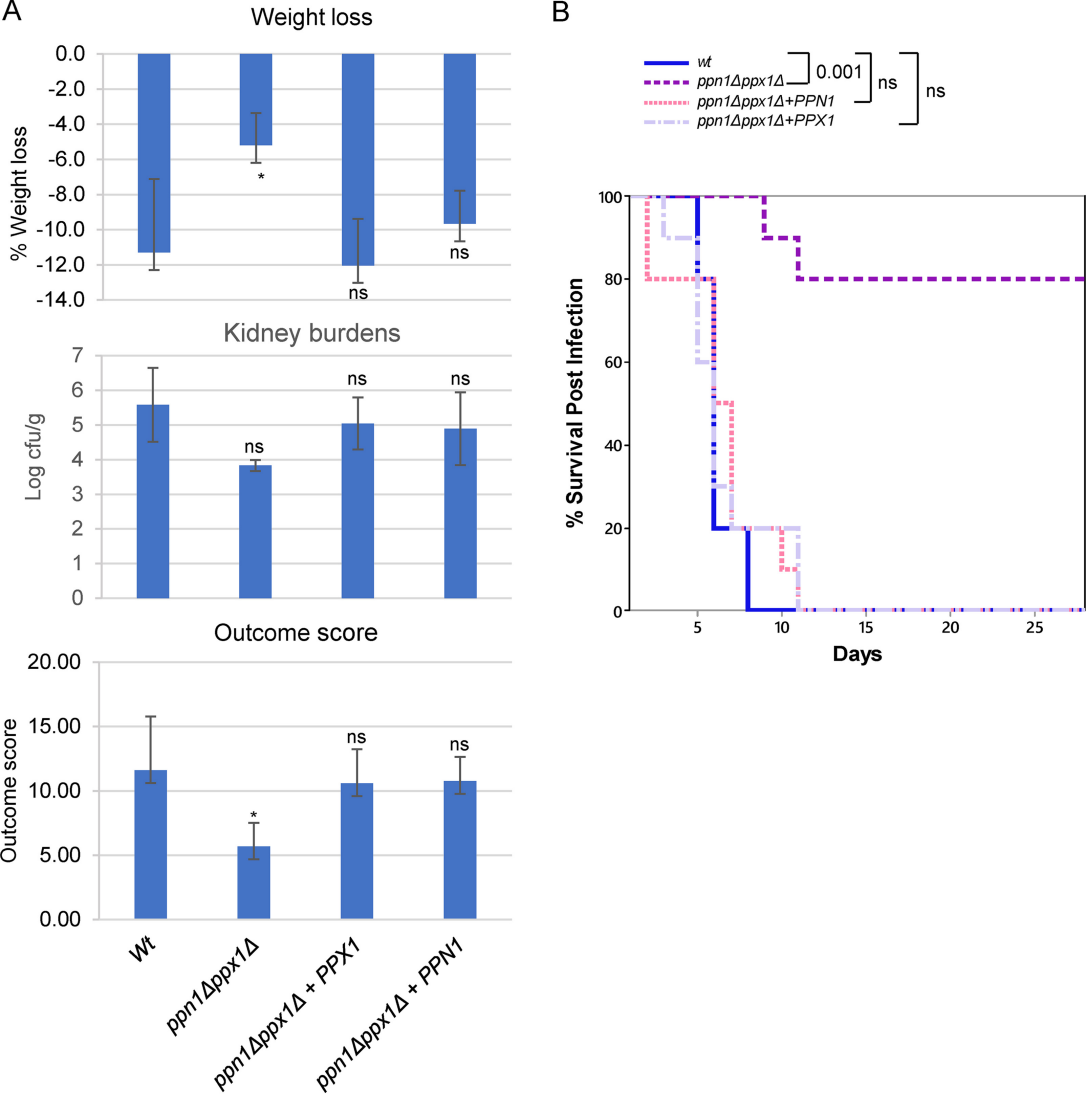
Virulence analysis in murine models of infection. (A) Three-day infection model. Kidney burden, percentage weight loss, and outcome scores for mice (*n* = 6) infected with the indicated strains. Comparison of WT, *ppn1*Δ *ppx1*Δ*+PPN1*, and *ppn1*Δ *ppx1*Δ*+PPX1* strain-infected mice with *ppn1*Δ *ppx1*Δ strain-infected mice by Kruskal-Wallis statistical analysis demonstrated a significant difference with *ppn1*Δ *ppx1*Δ strain-infected mice across all three parameters. ns, not significant; *, *P* < 0.05. (B) Twenty-eight-day survival model. Mice were injected with the same strains as for panel A, and survival was monitored daily. Survival curves were created using 10 mice per group except for the *ppn1*Δ *ppx1*Δ*+PPX1* strain (*n* = 9). Comparing survival of each strain with WT cells, only *ppn1*Δ *ppx1*Δ cells were highly significantly different (Kruskal-Wallis nonparametric test).

Following on from the observation that cells lacking Ppn1 and Ppx1 have a dramatic effect on C. albicans virulence in G. mellonella, the virulence of *ppn1*Δ *ppx1*Δ cells was examined in murine models of systemic candidiasis. The 3-day murine intravenous challenge model of C. albicans infection (10) combines weight loss and kidney fungal burden measurements after 72 h of infection to give an outcome score calculated as log(renal CFU/gram) − (0.5 × percent weight change), with a higher score indicating greater virulence. Mice infected with *ppn1*Δ *ppx1*Δ cells had a significantly lower kidney fungal burden and weight loss than those infected with wild-type cells, which culminated in a significantly lower outcome score (*P* < 0.01) (Fig. 7A). This virulence defect was restored by reintroduction of either *PPN1* or *PPX1*, which resulted in kidney fungal burdens, weight loss, and thus outcome scores similar to those seen with wild-type cells (Fig. 7A). The same strains were examined in the 28-day survival model of systemic candidiasis (Fig. 7B). Mice injected with *ppn1*Δ *ppx1*Δ cells survived significantly longer than mice injected with wild-type cells or reconstituted strains expressing either *PPN1* or *PPX1*. Mean survival times were 6.2 ± 0.3 days for wild-type (WT), 6.0 ± 0.7 for *ppn1*Δ *ppx1*Δ*+PPX1*, 7.4 ± 0.5 for *ppn1*Δ *ppx1*Δ*+PPN1*, and 19.8 ± 2.4 for *ppn1*Δ *ppx1*Δ cells (means ± standard errors of the means [SEM]). Therefore, cells lacking *PPN1* and *PPX1* display significantly attenuated virulence in both 3-day and 28-day murine models of systemic candidiasis which mirrors that seen in the G. mellonella model (Fig. 6). However, in both murine models of infection, reintroduction of either *PPN1* or *PPX1* restored wild-type levels of virulence to *ppn1*Δ *ppx1*Δ cells, thus indicating that Ppn1 and Ppx1 function redundantly to promote C. albicans virulence.

## DISCUSSION

**FIG 8:**
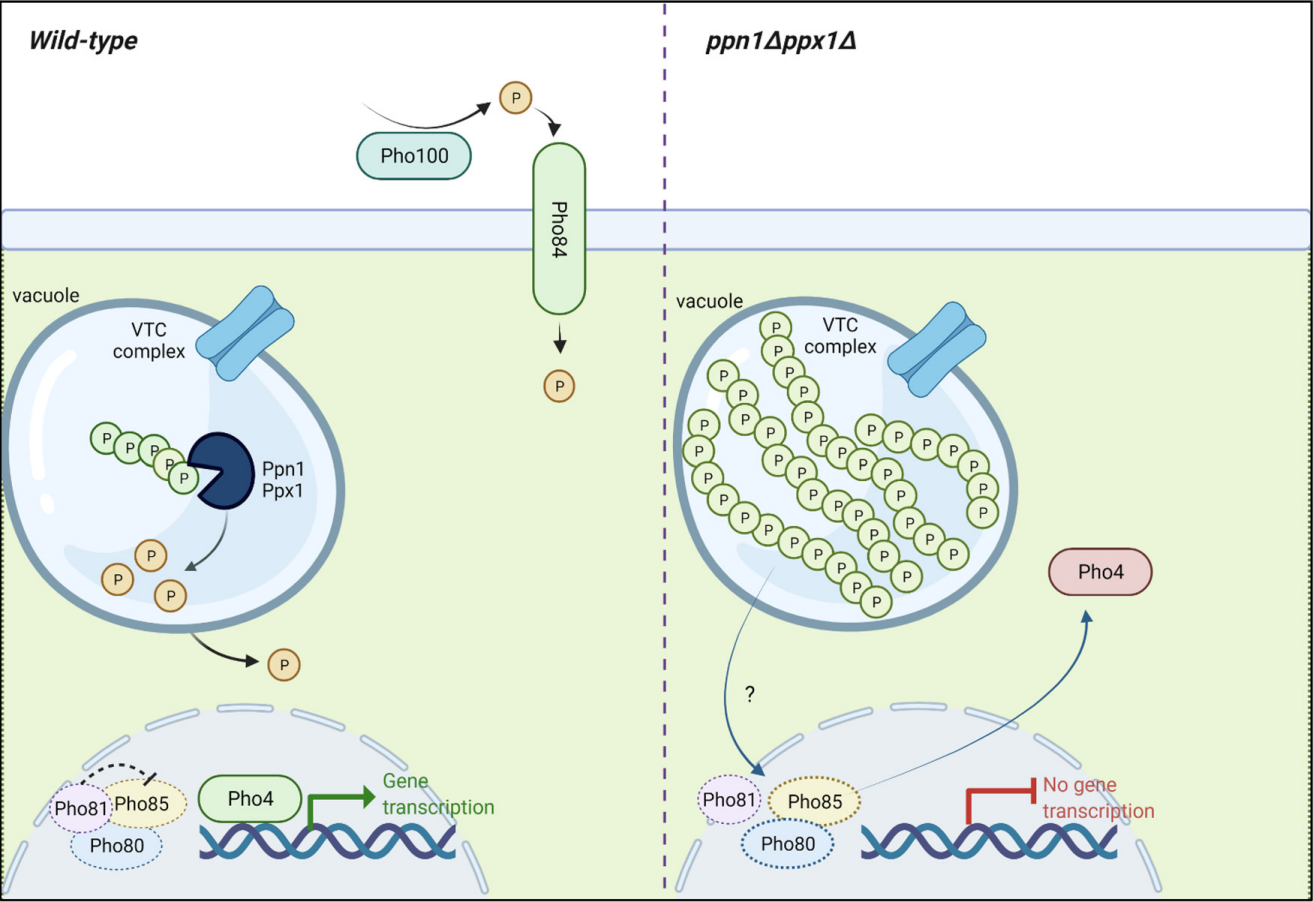
Activation of Pho4 is inhibited when polyP mobilization is hindered. Growth of wild-type C. albicans cells in P_i_-limiting medium stimulates polyP mobilization, the nuclear accumulation of Pho4, and the induction of Pho4-dependent genes with roles in P_i_ acquisition. However, in cells lacking the Ppn1 and Ppx1 polyphosphatases, polyP mobilization is dramatically impaired following growth in P_i_-limiting medium. Furthermore, Pho4 fails to accumulate in the nucleus and activate P_i_ acquisition genes. This suggests that an inability to mobilize polyP in C. albicans prevents the activation of Pho4. In S. cerevisiae, Pho4 is activated following P_i_ limitation via Pho81 inhibition of the Pho80-Pho85 cyclin-CDK complex, which negatively regulates Pho4. It is unknown if C. albicans Pho4 is regulated in the same way (as indicated by the dashed lines), but it is possible that polyP presence interferes with mechanism by which the cell senses P_i_-limiting environments. The figure was created with BioRender.com.

Here, we show that two polyphosphatases, Ppn1 and Ppx1, function redundantly to mobilize polyP stores in C. albicans. Moreover, we find that blocking polyP mobilization impairs PHO pathway activation, stress resistance, and morphogenetic switching (Fig. 8). Consistent with these traits being important for the pathobiology of C. albicans, cells lacking Ppx1 and Ppn1 display significantly attenuated virulence in both G. mellonella and murine models of infection.

The functional redundancy that exists between Ppn1 and Ppx1 in C. albicans was unanticipated, as in S. cerevisiae, deletion of Ppn1 alone results in the accumulation of longer-chain polyP molecules than in wild-type cells (22, 40). However, both Ppn1 and Ppx1 need to be deleted from the C. albicans genome before clear differences in polyP chain length and defects in polyP mobilization are seen. In S. cerevisiae, a total of four enzymes exhibiting polyphosphatase activity have been identified: Ppn1 (18), Ppn2 (41), Ppx1 (20), and Ddp1 (42). Only Ppn1 and Ppn2 are located in the vacuole, with Ppx1 and Dpd1 being predominantly cytoplasmic enzymes (40). Ppn1 and Ppn2 appear to be the major polyphosphatases in S. cerevisiae; deleting Ppn2 in *ppn1*Δ cells further increases the size of polyP chains (40). The C. albicans genome contains, in addition to Ppn1 and Ppx1 genes, homologues of Ppn2 (C7_03500W) and Ddp1 (C5_02220C) genes, which share 29% and 49% identity to their respective S. cerevisiae homologues. Thus, although we find that deletion of Ppn1 and Ppx1 in C. albicans largely prevents polyP mobilization, the small amount of mobilization seen in *ppn1*Δ *ppx1*Δ cells following P_i_ starvation may be due to Ppn2 or Ddp1 activity. Nonetheless, the functional redundancy that exists between Ppn1 and Ppx1 in C. albicans is clear; reintroduction of either *PPX1* or *PPN1* into *ppx1*Δ *ppn1*Δ cells rescues the myriad of phenotypes exhibited by the double mutant. This is particularly intriguing with regard to Ppx1; it is predicted to be a cytoplasmic enzyme, and yet the majority of polyP is stored in the vacuole.

In this study, we also found that blocking polyP mobilization impairs activation of the PHO pathway, and this, to the best of our knowledge, is the first such report (Fig. 8). Even following sustained growth under P_i_ starvation conditions (16 h), we saw no clear nuclear accumulation of Pho4 in *ppn1*Δ *ppx1*Δ cells and the induction of P_i_ acquisition genes was significantly impaired. This indicates that polyP presence (including polyP that cannot be mobilized) inhibits activation of the PHO pathway. The mechanism underlying this is unknown, although there have been recent breakthroughs in understanding how cells sense intracellular P_i_ levels (6). Many proteins involved in P_i_ homeostasis contain an SPX domain, which has been found to form a basic binding region for inositol pyrophosphate (PP-IP) ligands (43). As changes in P_i_ availability alter PP-IP levels (43), these molecules have been defined as “metabolic messengers” able to signal the P_i_ levels of the cell (44). In S. cerevisiae, the cyclin-dependent kinase (CDK) inhibitor Pho81 is regulated by PP-IP binding, which in turn regulates the activity of the Pho80-Pho85 cyclin-CDK complex; a negative regulator of the Pho4 transcription factor (45). Moreover, a recent study revealed that in the fungal pathogen Cryptococcus neoformans, PP-IP binding to Pho81 stabilizes the association of this CDK inhibitor with Pho80-Pho85 to promote PHO pathway activation and phosphate acquisition (46). In S. cerevisiae and C. albicans, polyP levels are also governed by PP-IPs, which bind to the SPX domain in Vtc4, stimulating its polyP-synthetase activity (43). Could the reverse also be true, i.e., polyP levels influence the levels of PP-IPs to inform the cell of P_i_ levels? In this scenario, the abundant yet unavailable polyP levels present in *ppn1*Δ *ppx1*Δ cells could signal to the cell that P_i_ levels are plentiful. This requires further investigation, but the observation that in C. albicans, as in S. cerevisiae (47, 48), the activation of Pho4-dependent genes occurs more rapidly in cells lacking polyP is consistent with the overall concept that polyP presence inhibits activation of P_i_ acquisition.

Consistent with Pho4 activation being compromised in cells that cannot mobilize polyP, *ppn1*Δ *ppx1*Δ cells display impaired growth on the same stresses that require Pho4 for resistance. However, cells lacking Pho4 are considerably more stress-sensitive than *ppn1*Δ *ppx1*Δ cells. This could be due to the fact that *pho4*Δ cells have no polyP and significantly lower P_i_ levels than wild-type cells (5), whereas there is residual polyphosphatase activity in *ppn1*Δ *ppx1*Δ cells.

Blocking polyP mobilization in C. albicans also results in significant morphological defects; both single *ppx1*Δ and *ppn1*Δ mutants are larger than wild-type cells, which is exacerbated in the double mutant, in which large swollen pseudohyphal cells are prevalent. Such morphological defects may be due in part to delays in cell cycle progression, as a recent study in S. cerevisiae revealed that P_i_ is mobilized from polyP to support dNTP synthesis and normal S-phase progression (25). Consistent with this, we found that C. albicans cells lacking both Ppn1 and Ppx1 are extremely sensitive to hydroxyurea, which blocks dNTP synthesis. Moreover, as Pho4 regulation is impaired in *ppn1*Δ *ppx1*Δ cells, it is noteworthy that “cell cycle” is one of the GO biological processes that are deregulated in *pho4*Δ cells compared with wild-type cells (5). A further morphological facet of *ppn1*Δ *ppx1*Δ cells is their impaired ability to form true hyphae following growth at 37°C in the presence of serum. PolyP mobilization does not appear to be stimulated during the yeast-to-hypha transition (Y. Ahmed and J. Quinn, unpublished data); thus, the inability of *ppn1*Δ *ppx1*Δ cells to form true hyphae may instead be linked to the cell cycle/morphological defects characteristic of this mutant. It is noteworthy, in this regard, that in a fungal pathogen of maize, Ustilago maydis, polyP is important for filamentous growth (49).

As we show that preventing polyP mobilization impacts the virulence of C. albicans, this study adds to a growing body of evidence that P_i_ homeostasis is important for the pathogenesis of this major human pathogen. Previous studies have shown that the Pho4 transcription factor is important for survival of C. albicans following phagocytosis and in systemic and commensal models of infection (5, 30), and deletion of the major phosphate transporter Pho84 also significantly impairs virulence (9), with a recent study revealing that Pho84 also promotes fungal commensalism (50). In this study, we show that preventing polyP mobilization impairs important pathogenesis traits, including PHO pathway activation and the yeast-to-hypha switch, which likely contribute to the virulence defect in *ppn1*Δ *ppx1*Δ cells. Indeed, the PHO pathway targets, *PHO84* and *PHO100*, which are downregulated in *ppn1*Δ *ppx1*Δ cells, are both required for C. albicans virulence (9, 10). We also provide the first evidence that polyP presence contributes to the pathogenesis of C. albicans, as cells lacking the Vtc4 polyP synthase display impaired virulence in G. mellonella, albeit not to the same extent as in *ppn1*Δ *ppx1*Δ cells. Other studies have shown that P_i_ homeostasis is important for the virulence of a further major human fungal pathogen, Cryptococcus neoformans. Phosphate acquisition is essential, as cells lacking the Pho4 transcription factor were hypovirulent in both intranasal and intravenous models of cryptococcosis (51), and in a separate study, deletion of three phosphate transporters also resulted in reduced virulence (52). In the latter study, C. neoformans cells lacking homologues of the Ppn1 and Ppx1 polyphosphatases, Epp1 and Xpp1, were also created. While the double *epp1*Δ *xpp1*Δ mutant had higher levels of polyP than wild-type cells, this strain behaved like wild-type cells in a macrophage interaction assay (52). Hence, it would be interesting to explore if, as reported here for C. albicans, PHO pathway activation is likewise attenuated in C. neoformans cells lacking Epp1 and Xpp1.

In summary, we have demonstrated that polyP mobilization plays a multifaceted role in the pathobiology of C. albicans. It is intriguing that blocking polyP mobilization in C. albicans results in much stronger phenotypes than actually preventing the synthesis of this polymer. This may be due to the fact that blocking polyP mobilization impairs activation of the PHO pathway and therefore acquisition of extracellular P_i_. P_i_ acquisition is an important virulence trait in several pathogens, and P_i_ homeostasis mechanisms differ significantly between fungal pathogen and host, making this an excellent target for new antifungal drug discovery.

## MATERIALS AND METHODS

### Media.

All strains were grown at 30°C in YPD medium (2% Bacto peptone, 1% Bacto yeast extract, 2% glucose) unless otherwise stated. For P_i_-limiting conditions, strains were either grown in low-phosphate YPD medium (YPD-LPi) (2% Bacto peptone, 1% yeast extract base without phosphate [ForMedium], 2% glucose) or PNMC (peptone, 2.5 g/L; NaCl, 3 g/L; MgSO_4_, 1 mM; CaCl_2_, 1 mM) (53) supplemented with 20% glucose. As Bacto peptone contains 0.4% P_i_, this equates to 0.8 mM P_i_ in YPD-LPi and 0.1 mM P_i_ in PNMC. Both YPD-LPi and PNMC were supplemented with 10 mM KH_2_PO_4_ to generate YPD+P_i_ and PNMC+P_i_, respectively.

### Strain construction.

All strains used in this study are listed in Table 1, and oligonucleotides are in Table S1.

**TABLE 1:** Strains used in this study

Strain	Name	Genotype	Reference or source
SN148		*arg4*Δ/*arg4*Δ *leu2*Δ/*leu2*Δ *his1*Δ/*his1*Δ *ura3*Δ::*imm434*/*ura3*Δ::*imm434 iro1*Δ::*imm434*/*iro1*Δ::*imm434*	55
JC747	WT *SN148 + CIp30*	*arg4 leu2*/*leu2 his1*/*his1 ura3*::λ*imm434*/*ura3*::λ*imm434 iro1*::λ*imm434*/*iro1*::λ*imm434 CIp10*	59
JC1936	WT	*SN152 arg4*Δ/*arg4*Δ *leu2*Δ/*leu2*Δ *his1*Δ/*his1*Δ *ura3*/*ura3*Δ::*imm434 IRO1*/*iro1*Δ::*imm434 CIp10*	5
JC1928	*pho4*Δ	*SN152 arg4*Δ/*arg4*Δ *leu2*Δ/*leu2*Δ *his1*Δ/*his1*Δ *ura3*/*ura3*Δ::*imm434 IRO1*/*iro1*Δ::*imm434 pho4*Δ::*HIS1*/*pho4*Δ::*LEU2 CIp10*	5
JC1917	*pho4*Δ *+ CIp10-PHO4*	*SN152 arg4*Δ/*arg4*Δ *leu2*Δ/*leu2*Δ *his1*Δ/*his1*Δ *ura3*/*ura3*Δ::*imm434 IRO1*/*iro1*Δ::*imm434 pho4*Δ::*HIS1*/*pho4*Δ::*LEU2 CIp10-PHO4*	5
JC1977	*PHO4-GFP*	*SN148 pACT-PHO4-GFP:URA3*	5
JC1984	*vtc4*Δ *+ CIp10*	*SN148 vtc4*::*loxP-ARG4-ura3-loxP*/*vtc4*::*loxP-HIS1-loxP CIp10*	5
JC2014	*vtc4*Δ *+ CIp10-VTC4*	*SN148 vtc4*::*loxP-ARG4-ura3-loxP*/*vtc4*::*loxP-HIS1-loxP CIp10-VTC4*	5
JC1991	*ppx1*Δ *+ CIp10*	*SN148 ppx1*::*loxP-ARG4-ura3-loxP*/*ppx1*::*loxP-HIS1-loxP CIp10*	This work
JC1985	*ppn1*Δ *+ CIp10*	*SN148 ppn1*::*loxP-ARG4-ura3-loxP*/*ppn1*::*loxP-HIS1-loxP CIp10*	This work
JC2016	*ppn1*Δ *+ CIp10-PPN1*	*SN148 ppn1*::*loxP-ARG4-ura3-loxP*/*ppn1*::*loxP-HIS1-loxP CIp10-PPN1*	This work
JC2283	*ppx1*Δ *+ CIp10-PPX1*	*SN148 ppx1*::*loxP-ARG4-ura3-loxP*/*ppx1*::*loxP-HIS1-loxP CIp10-PPX1*	This work
JC2210	*ppn1*Δ *ppx1*Δ *+ CIp10*	*SN148 ppn1*::*loxP-ARG4-ura3-loxP*/*ppn1*::*loxP-HIS1-loxP ppx1*Δ::*loxP*/*ppx1*Δ::*loxP CIp10*	This work
JC2257	*ppn1*Δ *ppx1*Δ *+ CIp10-PPN1*	*SN148 ppn1*::*loxP-ARG4-ura3-loxP*/*ppn1*::*loxP-HIS1-loxP ppx1*Δ::*loxP*/*ppx1*Δ::*loxP CIp10-PPN1*	This work
JC2267	*ppn1*Δ *ppx1*Δ *+ CIp10-PPX1*	*SN148 ppn1*::*loxP-ARG4-ura3-loxP*/*ppn1*::*loxP-HIS1-loxP ppx1*Δ::*loxP*/*ppx1*Δ::*loxP CIp10-PPX1*	This work
JC2303	*ppn1*Δ *ppx1*Δ *+ PHO4-GFP*	*SN148 ppn1*::*loxP-ARG4-ura3-loxP*/*ppn1*::*loxP-HIS1-loxP ppx1*Δ::*loxP*/*ppx1*Δ::*loxP*/*pACT-PHO4-GFP*:*URA3*	This work

10.1128/mbio.00342-22.6 TABLE S1 Oligonucleotides used in this study. Download Table S1, DOCX file, 0.02 MB. Copyright © 2022 Ahmed et al. 2022 Ahmed et al. https://creativecommons.org/licenses/by/4.0/ This content is distributed under the terms of the Creative Commons Attribution 4.0 International license.

### Deletion of *PPN1* and *PPX1*.

To delete *PPN1*, disruption cassettes containing *ARG4* or *HIS1* nutritional marker genes, flanked by *loxP* sites and 91 bp 5′ and 3′ of the *PPN1* open reading frame (ORF), were generated by PCR using oligonucleotide primers Ppn1delF and Ppn1delR and the plasmid template pLAL or pLHL (54). Following amplification, deletion cassettes were sequentially transformed into SN148 wild-type cells (55) to disrupt both alleles of *PPN1*. The same strategy was used to disrupt both alleles of *PPX1*, using the oligonucleotides Ppx1delF and Ppx1delR to create the *ppx1*Δ strain. PCR was used to confirm disruption of each allele. Uridine prototrophy was restored by integrating Clp10 at the *RSP1* locus. This generated the *ppn1*Δ (JC1991) and *ppx1*Δ (JC1985) strains. To reintegrate *PPN1* into *ppn1*Δ cells, the ORF and the promoter and terminator regions were amplified by PCR using the oligonucleotide pair PPN1CIFBamHI and PPN1CIRBamHI and ligated into the BamHI site of Clp10 to generate CIp10-*PPN1*. Clp10-*PPN1* was linearized with StuI to integrate at the *RPS1* locus of *ppn1*Δ cells, generating the *ppn1*Δ+*PPN1* strain (JC2016). The same strategy but with the oligonucleotides PPX1CIfBamHI and PPX1CIRBamHI was used to create the *ppx1*Δ+*PPX1* strain (JC2283).

To delete both *PPX1* and *PPN1*, the Clox system with nourseothricin selection was used (56). A *PPX1* disruption cassette containing the NAT1-Clox marker gene was PCR amplified using the oligonucleotides Ppx1natDelF and Ppx1natDelR. This was sequentially transformed into *ppn1*Δ cells to disrupt both alleles of *PPX1*, generating the *ppn1*Δ *ppx1*Δ mutant. CIp10 was integrated at the *RSP1* locus to generate *ppn1*Δ *ppx1*Δ (JC2210). To reintegrate either *PPN1* or *PPX1*, CIp10-*PPN1* and CIp10-*PPX1* were linearized with StuI to integrate at the *RPS1* locus of *ppn1*Δ *ppx1*Δ cells, generating *ppn1*Δ *ppx1*Δ+*PPN1* (JC2257) and *ppn1*Δ *ppx1*Δ+*PPX1* (JC2267) cells, respectively. To facilitate localization of Pho4 in *ppn1*Δ *ppx1*Δ cells, the plasmid pACT-PHO4GFP (5) was linearized with StuI and transformed into *ppn1*Δ *ppx1*Δ cells, generating JC2303.

### Polyphosphate analysis.

Intracellular polyP granules were examined by Neisser staining (31) and visualized by light microscopy. Cells grown in PNMC were fixed with paraformaldehyde (57), mounted on a slide, and stained with solution A (methylene blue, 0.1%; glacial acetic acid, 5%; ethanol, 5%) and solution B (crystal violet, 10%) for 15 s. Slides were stringently rinsed with water and allowed to dry, followed by staining with solution C (chrysoidin Y, 1%) for 45 s and stringent washing. Images were captured using a Zeiss Axioscope (differential inference contrast [DIC] setting) with a 63× oil immersion objective.

For urea-PAGE analysis, RNA and polyP were extracted as previously described (5). Twenty micrograms of RNA containing polyP was resolved on 12% polyacrylamide TBE-urea gels (Bio-Rad) in 1× Tris-borate-EDTA (TBE) buffer. Following electrophoresis, gels were fixed with glycerol and methanol, stained with toluidine blue, and destained as described previously (5).

### ICP-MS.

Exponentially growing cells, grown in YPD at 30°C, were harvested by centrifugation, washed twice with 25 mL of Tris buffer (50 mM Tris, pH 7.5), incubated in the same buffer containing 10 mM EDTA for 5 min at room temperature to remove surface-bound metal, and then washed twice with 25 mL of the same buffer without EDTA. Washed pellets were digested in 1 mL of 65% (wt/vol) HNO_3_ (Merck) and incubated for >48 h at room temperature. The triplicate digested samples were centrifuged (13,000 × *g*, 20 min), and the supernatants were diluted 1:10 with 2% (wt/vol) HNO_3_ solution, which contained 20 μg/L Ag and Pt as internal standards, and analyzed by inductively coupled plasma mass spectrometry (ICP-MS) essentially as previously described (58). Differences were tested for statistical significance by one-way analysis of variance (ANOVA).

### Yeast-hypha switch assay.

Hyphal formation was induced by diluting stationary-phase cells 1:10 in YPD medium containing 10% fetal calf serum (FCS) and incubated at 37°C for 3 h. DIC images were captured using a Zeiss Axioscope with a 63× oil immersion objective.

### Spot tests.

Overnight C. albicans cultures grown in YPD were diluted back to an optical density at 660 nm (OD_660_) of 0.2 and then grown to mid-exponential phase (OD_660_ ≈ 0.7) before being diluted back to an OD_660_ of 0.2 in fresh YPD or YPD-LPi, and serial 10-fold dilutions were spotted onto YPD or YPD-LPi agar plates containing the specified stress-inducing compounds using a 48-well replica plater (Sigma). Plates were incubated for 24 to 48 h at 30°C.

### Cell volume and growth analysis.

Cell volume was measured using a Beckman cell counter and analyzer system via pulse area analysis. Cells were grown to an equal OD_660_ of 0.7 before being sonicated (35 kHz, 15 s) to negate any artifacts cause by cells clumping together. From this, 200 μL of sample was added to 10 mL of filtered CASYton in sterile CASY cups. Samples were mixed immediately prior to measurement. The Beckman cell counter and analysis system was washed 3 times prior to sample analysis and checked for background. A wash step was performed between each sample read. Student’s two-sample *t* test was used to investigate whether the cell volume of specified strains differed from that of the wild type.

Growth analysis was performed as follows. Overnight cultures were grown in YPD and diluted back to a starting OD_660_ of 0.1. Samples were taken at time zero from the OD_660_ 0.1 culture, which included an OD reading and a cell count using a hemocytometer. This was repeated hourly.

### Pho4-GFP localization and Western blotting.

Wild-type and *ppn1*Δ *ppx1*Δ cells expressing Pho4-GFP (5) were grown in YPD, a sample was removed (*t* = 0), and then the remaining cells were washed 3 times, resuspended in YPD-LPi, and grown for the indicated times. Samples were processed as previously described (57). GFP and DAPI fluorescence was captured with a Zeiss AxioImager with a 63× oil immersion objective (Newcastle University Bioimaging Facility). Protein samples were subjected to electrophoresis on 8% SDS polyacrylamide gels and transferred to a nitrocellulose membrane. Membranes were blocked in 10% bovine albumin serum (BSA) in TBST (1 mM Tris-HCl [pH 8], 15 mM NaCl, 0.1% Tween 20 [vol/vol]) at room temperature for 30 min with gentle agitation. Following blocking, membranes were incubated with an anti-GFP antibody (Sigma, Dorset, UK) overnight at 4°C with gentle agitation. Membranes were subsequently washed 3 times in TBST before being incubated with a horseradish peroxidase (HRP)-conjugated anti-mouse secondary antibody (Sigma, Dorset, UK) for 1 h at room temperature. Development of membranes was carried out manually using an ECL Western blot detection system (Amersham Pharma Biotech) and Fuji Medical X-ray film. Tubulin was used as a loading control using an anti-tubulin monoclonal primary antibody (DSHB, University of Iowa) and the secondary antibody described above.

### Hyperpolarized bud stimulation.

Hyperpolarized bud formation was induced by diluting stationary-phase cells grown overnight 1:10 in fresh YPD liquid medium containing 40 mM HU and incubating at 30°C for 6 h at 180 rpm. Cells were fixed with 3.7% paraformaldehyde, and images were captured using a Zeiss Axioscope with a 63× oil immersion objective. For each strain, 200 cells were measured using Zeiss imaging software. Analysis was carried out to determine the means and standard deviations (SD). Statistical analysis was performed using Student’s two-sample *t* test.

### Vacuolar staining.

For vacuolar staining, prior to fixing, cells were washed 3 times in phosphate-buffered saline (PBS) to remove traces of YPD, as this can interfere with the dye. Cultures were incubated with 100 mM 7-amino-4-chloromethylcoumarin (CMAC) for 30 min in the dark followed by fixation with 3.7% paraformaldehyde. CAMC-stained images were captured using a Zeiss Axioscope by excitation at 460 nm.

### RNA extraction.

Cells were grown to mid-log phase in at 30°C YPD prior to being harvested, washed twice in YPD-LPi, and then resuspended in YPD-LPi. Samples were collected following 0, 4, and 16 h growth in YPD-LPi, washed twice in ice-cold H_2_O, and then snap-frozen in liquid nitrogen.

For extraction, pellets were thawed on ice before being resuspended in 750 μL TES (10 mM Tris-HCl [pH 7.5], 5 mM EDTA [pH 7.5], 1% SDS [wt/vol]) and 750 μL of acidic phenol-chloroform. Following this, samples were incubated at 65°C for 1 h with vortexing every 10 min. Subsequently, samples were incubated on ice for 1 min and centrifuged at 6,000 rpm for 15 min 4°C to separate the aqueous layer. To the aqueous layer, 700 μL of acidic phenol-chloroform was added and mixed by inversion before undergoing centrifugation at 13,000 rpm for 5 min 4°C to separate the aqueous layer. Following this, 700 μL of phenol-chloroform was added to the aqueous layer, mixed by inversion, and centrifuged at 13,000 rpm for 5 min at 4°C. To the aqueous layer, 2 volumes of 100% ethanol and 1/10 volume 3 M sodium acetate (NaAc) (pH 7) were added, and samples were incubated overnight at −80°C to precipitate the RNA. Following incubation, samples were pelleted at 13,000 rpm for 15 min at 4°C. Pellets were washed in 400 μL 70% ethanol, centrifuged at 13,000 rpm for 5 min at 4°C, and then resuspended in 50 μL of sterile nano-H_2_O. Samples were stored at −80°C until use.

### RT-qPCR.

For real-time qPCR, samples extracted were diluted according to their concentration to be used as a template for one-step reactions using the SuperScript III Platinum one-step qRT-PCR kit (Thermo Fisher) in 96-well plates run on an ABI machine. Threshold cycle (*C_T_*) values were determined using ABI software. For the tested targets, the single enrichment for each target was calculated using the comparative *C_T_* method. Actin was used for normalization using the primers ACT1F and ACT1R. RT-qPCR analysis was performed on transcripts of PHO pathway targets, *PHO84* (PHO84F and PHO84R) and *PHO100* (PHO100F and PHO100R), of 3 biological replicates in technical duplicate. Statistical significance was determined using ANOVA with Dunnett’s posttest for multiple comparisons.

### G. mellonella virulence assay.

To investigate C. albicans virulence, cultures were grown to mid-exponential phase (OD_660_ ≈ 0.7) and washed 3 times in PBS. Following this, 5 × 10^5^ cells were injected directly into the hemocoel via the last left proleg of 15 *Galleria* larvae. As a control, sterile PBS was injected into a further 15 larvae. Survival was assayed over a 5-day period at 37°C and is represented by Kaplan-Meier curves and analyzed by a log-rank test.

### Murine virulence assays.

Sixty-four female BALB/c mice (6 to 8 weeks old) were purchased from Envigo Ltd. and were allowed to acclimatize for 1 week in the animal facility. Mice were randomly assigned to 12 cages, eight with five mice each and four with six mice each. Mice were allowed free access to food and water throughout the study. Procedures were carried out under UK Home Office project license 70/9027 awarded to Donna MacCallum and were carried out by a UK Home Office personal license holder.

C. albicans strains were grown for 16 to 24 h in 0.1% Neopeptone, 0.4% glucose, 0.1% yeast extract (NGT) medium at 30°C at 200 rpm. Cells were harvested, washed twice in saline, and enumerated using a hemocytometer. For each strain, 10 mice were injected in the tail vein with 3.5 × 10^4^ CFU/g body weight in 100 μL sterile saline. Inoculum level was confirmed by viable-cell counting on Sabouraud dextrose agar. Inocula were randomly assigned to sets of cages, and strains were coded so that the researcher was blind to strain identity.

For the 3-day outcome experiment, the 6 mice in one cage were culled, weight change from day 0 to 3 was determined, and kidneys and brain were used to determine fungal burdens. Outcome score was determined using weight change and kidney burdens (10).

For the survival experiment, 10 mice (2 cages of 5 mice) were monitored over 28 days. Mice were weighed and monitored daily, with more frequent monitoring if mice became ill. Mice were culled if they lost 20% of their initial body weight, along with exhibiting ruffled fur and hunched posture, or if they developed a severe head tilt which affected their ability to reach food and water. Culled mice were recorded as having “died” on the following day. After 28 days, any surviving mice were culled. Kidneys and brains were removed for organ burden determination.

Results were compared in IBM SPSS version 25 using Kaplan-Meier log rank statistics to compare survival and the Kruskal-Wallis test (multiple groups) and Mann-Whitney U test (pairwise comparisons) for weight change, organ burdens, and outcome score comparisons. Surviving mice were considered censored data in survival curve statistics.
