# Supplementary figures and images for: Blocking Polyphosphate Mobilization Inhibits Pho4 Activation and Virulence in the Pathogen Candida albicans
Source: mBio. 2022 May 16;13(3):e00342-22. doi: 10.1128/mbio.00342-22 (PMC9239153; doi:10.1128/mbio.00342-22)

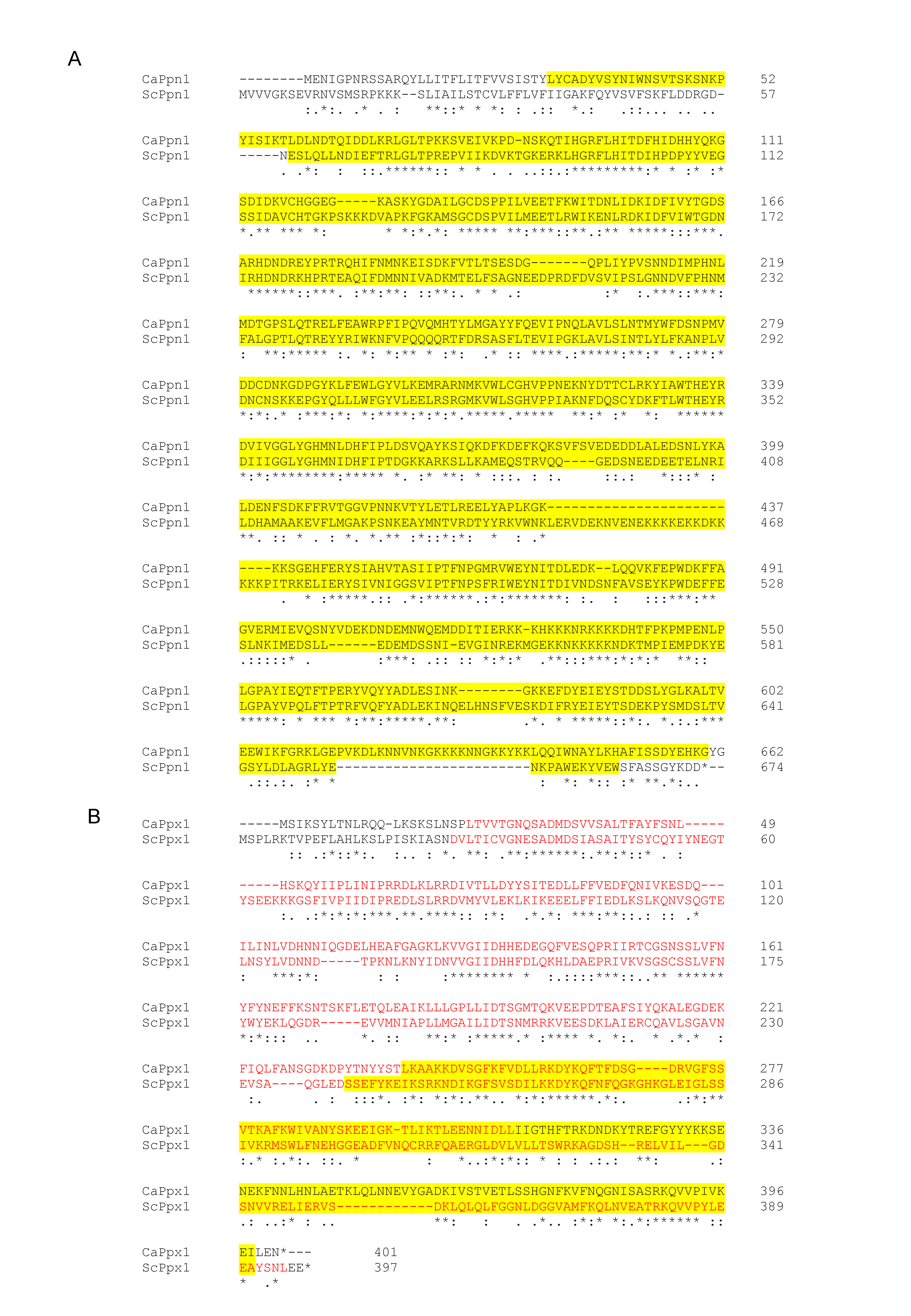

Supplement: FIG S1 [file mbio.00342-22-s0002.tif]

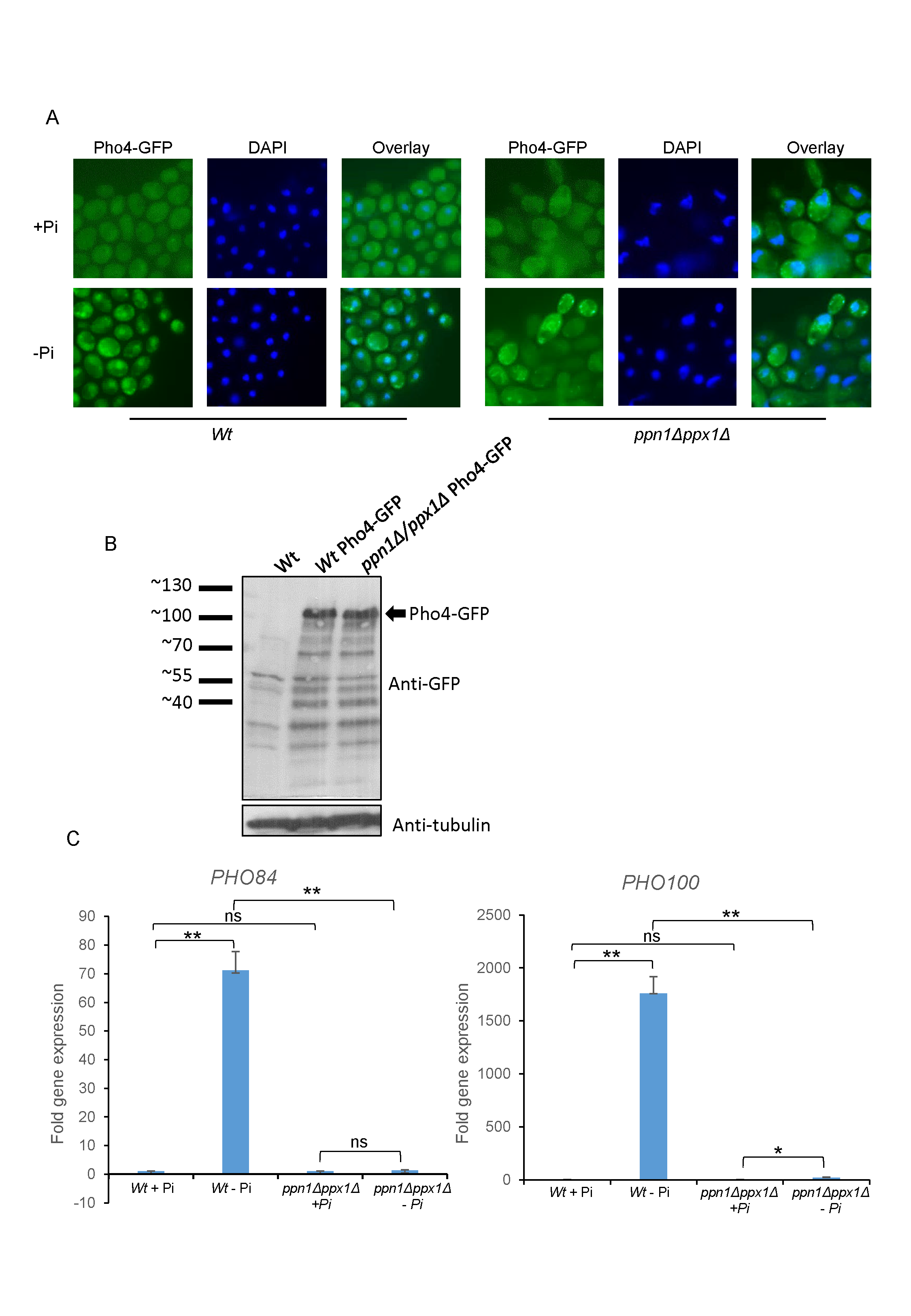

Supplement: FIG S2 [file mbio.00342-22-s0003.tif]

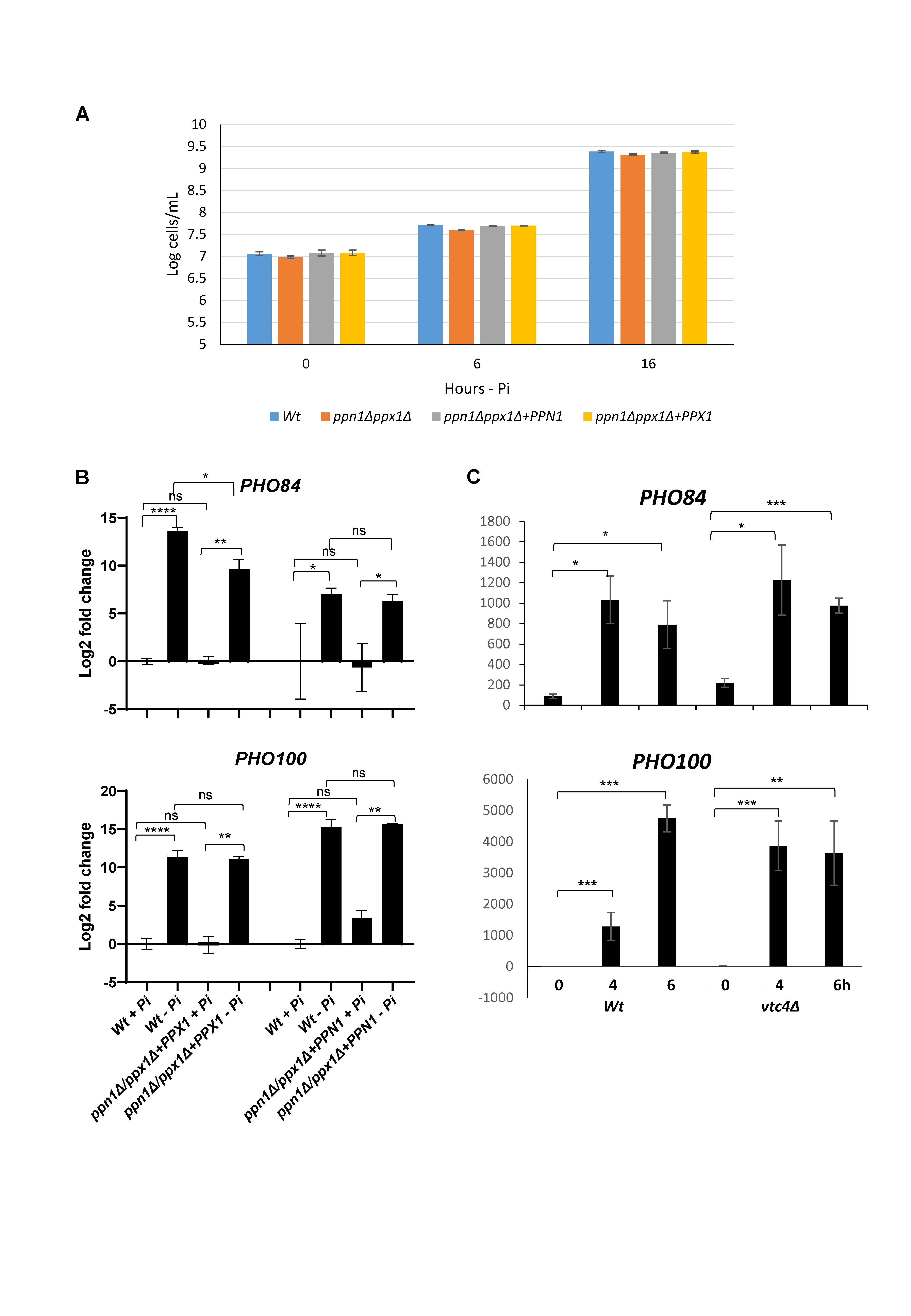

Supplement: FIG S3 [file mbio.00342-22-s0004.tif]

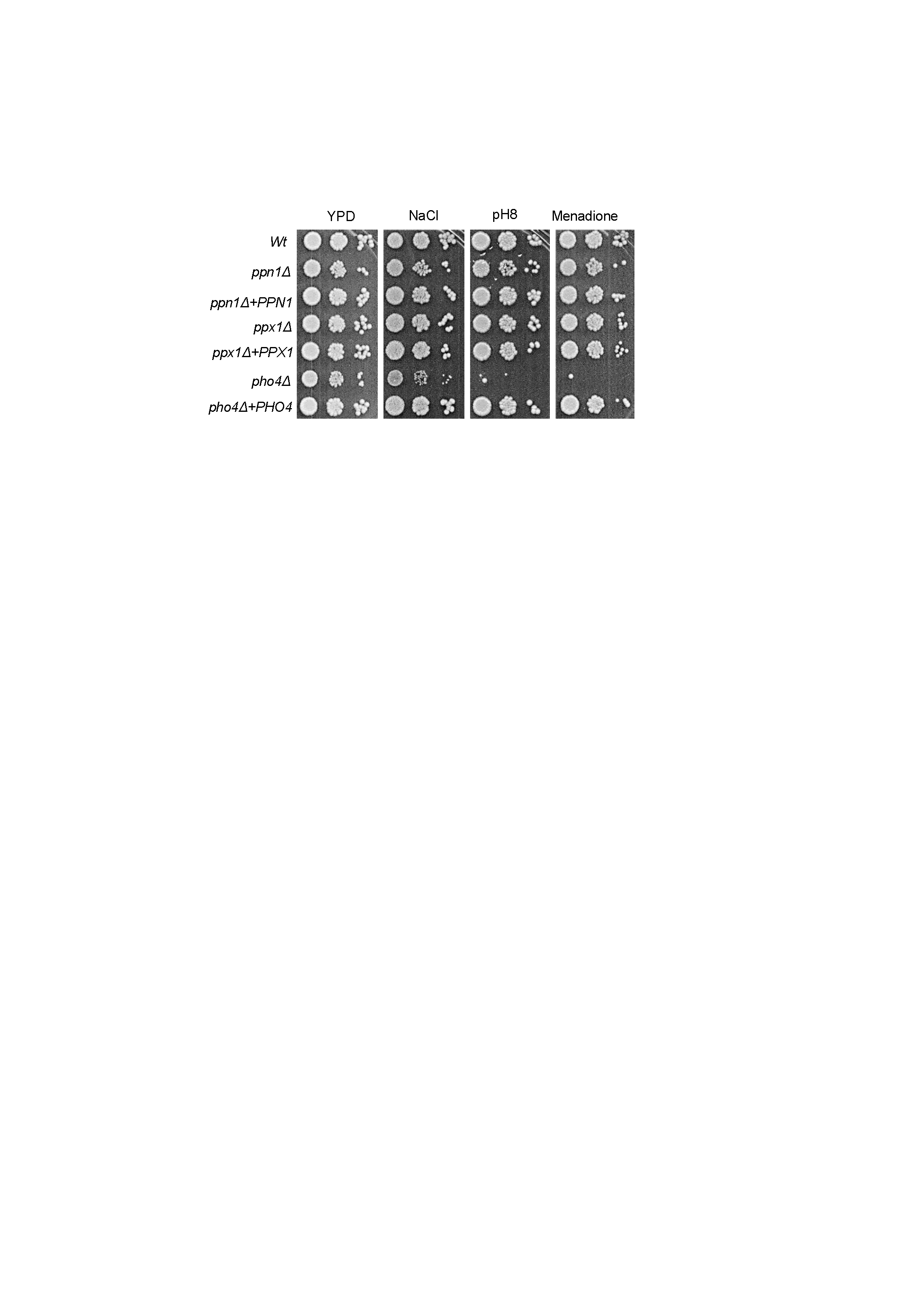

Supplement: FIG S4 [file mbio.00342-22-s0005.tif]

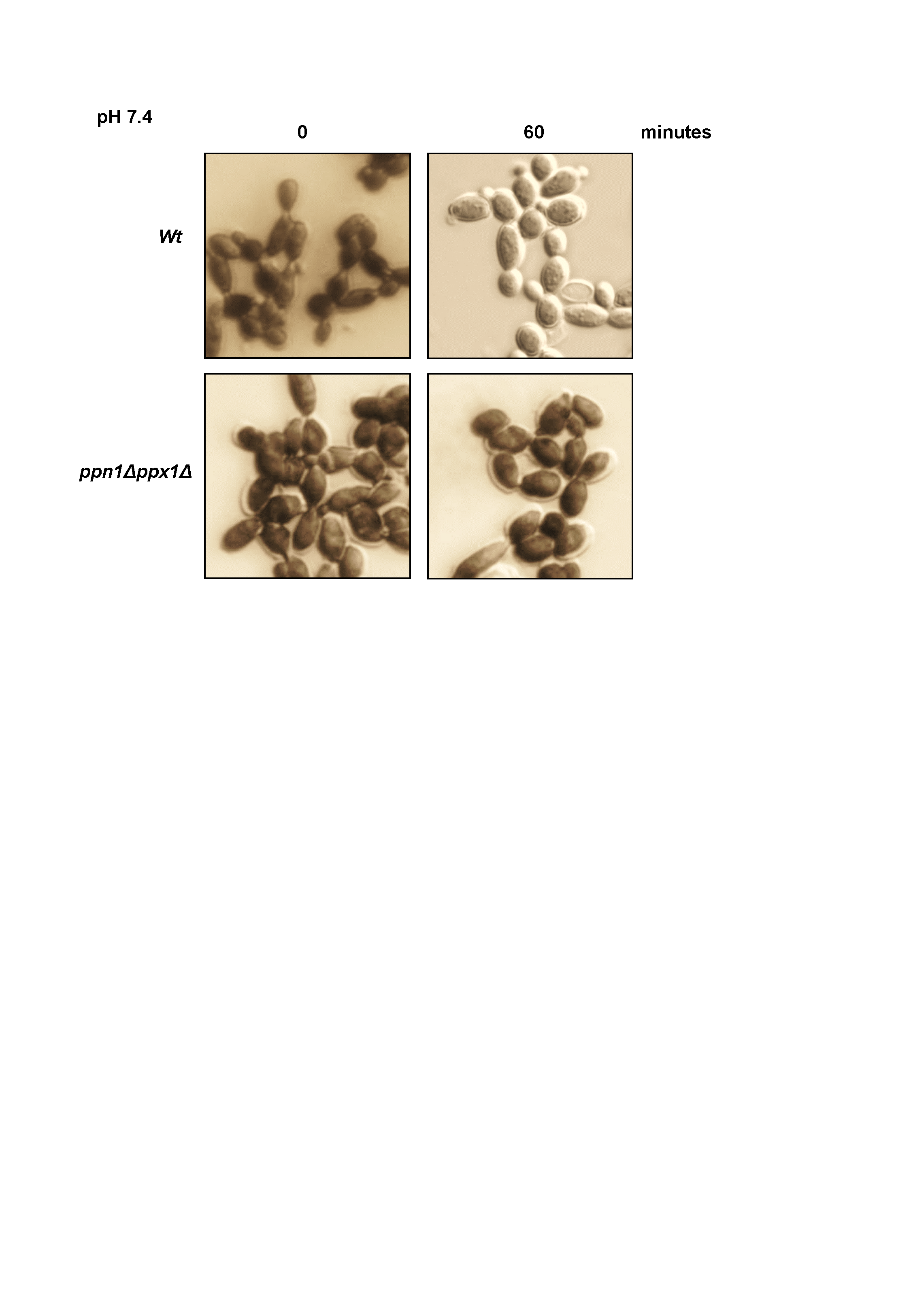

Supplement: FIG S5 [file mbio.00342-22-s0006.tif]
