# Supplementary material for: Blocking Polyphosphate Mobilization Inhibits Pho4 Activation and Virulence in the Pathogen Candida albicans
Source: mBio. 2022 May 16;13(3):e00342-22. doi: 10.1128/mbio.00342-22 (PMC9239153; doi:10.1128/mbio.00342-22)
Supplement: TABLE S1 [file mbio.00342-22-s0001.docx]

Table S1. Oligonucleotides used in this study

| **Oligonucleotide** | **Sequence 5’-3’** |
| --- | --- |
| PPX1natDelF | CGTCATACGTGAAGAGGAAGCACGTTCTATTTAAAGATATCTTCTCTTTTCATAAAAAATAAAAATCATCTCCAGTAGGGTTGTTCTTAATACTACAGTCACGGCCAGTGAATTGTAATA |
| PPX1natDel R | TATATTTCTGGTATTTGTCAAATTACGGCCAAAGATACTCTTCACATAGTTAACCATGACTATTAATAAATTAATCAATAGATTCGATGTGGATTCCAATTCGGAATTAACCCTCACTAA |
| Ppx1delF | CGTCATACGTGAAGAGGAAGCACGTTCTATTTAAAGATATCTTCTCTTTTCATAAAAAATAAAAATCATCTCCAGTAGGGTTGTTCTTAATACTACAGTCCCAGGGTTTTCCCAGTCAC |
| Ppx1delR | CATAAACAGTTTAATGCCGGTTTCTATGAGAAGTGTATCAATTGGTACTGATAATTATTTAATTAGTTATCTAAGCTACACCTAAGGTTATCAATTTCTCACTAAAGGGAACAAAAGC |
| PPN1delF | AACAATTAGATCCTTTTGGTTTTCATTTTCAATTCAATTTTTAGATTTTTTTTTATTTCATTTTTTTTTGTTTACTTATACATTTATATATTCAACATAACCAGGGTTTTCCCAGTCACG |
| PPN1delR | AAATGTAAAATTTATATTCGTTACTACATCAAAATAATCAAAAAATAAAAAAATAAAAAAATAGAATAATAAAAGTGACCATACAAAATAAAAATCGCTCACTAAAGGGAACAAAAGC |
| PPX1ChF | TTGTGGATATTTGTACCACGG |
| PPX1ChR | TTGGATGTTGTTGTGATCAAC |
| PPN1ChF | GCTTTACAACAATTTTATACTGG |
| PPN1ChR | GGATTCCAAATCGGCATAGTAT |
| PPX1CIFBamHI | GCGCGGATCCGCACCGATCCAAGAAACCAGTATG |
| PPX1CIRBamHI | GCGCGGATCCGGCTTAAATGTGGTGATTGATGGG |
| PPN1CIFBamHI | GCGCGGATCCGTACATTGTCATCGTGCATCC |
| PPN1CIRBamHI | GCGCGGATCCCAAGAAGTTAGACACCCTTGG |
| ARG R2 | CCCATCTAATAGGTTGAGC |
| HIS R2 | AATGGTTGCGTAATAAA |
| Pho4TagChF | CATGGAACGCCAATTTT |
| LOXPR | TTCGTATAATGTATGCTATACG |
| CycTermR | CGACAGCCATGTTGTAC |
| PHO84F | TTTGTTGGGTTTGTTCGTCA |
| PHO84R | GCAATAATGGCACCGACTTT |
| PHO100F | GCTGGCCACAATBTTTTCTT |
| PHO100R | ACAGCAGATGAGGCTGGAGT |
| ACT1F | ACCACCGGTATTGTTTTGGA |
| ACT1R | AGCGTAAATTGGAACAACGTG |
